# De novo genome assembly and transcriptome sequencing in foot and mantle tissues of *Megaustenia siamensis* reveals components of adhesive substances

**DOI:** 10.1038/s41598-024-64425-6

**Published:** 2024-06-14

**Authors:** Wanna Chetruengchai, Parin Jirapatrasilp, Chalurmpon Srichomthong, Adjima Assawapitaksakul, Arthit Pholyotha, Piyoros Tongkerd, Vorasuk Shotelersuk, Somsak Panha

**Affiliations:** 1 Center of Excellence for Medical Genomics, Department of Pediatrics, Faculty of Medicine, Chulalongkorn University, Bangkok, 10330 Thailand; 2 Excellence Center for Genomics and Precision Medicine, King Chulalongkorn Memorial Hospital, The Thai Red Cross Society, Bangkok, 10330 Thailand; 3 Animal Systematics Research Unit, Department of Biology, Faculty of Science, Chulalongkorn University, Bangkok, 10330 Thailand; 4 Academy of Science, The Royal Society of Thailand, Bangkok, 10300 Thailand

**Keywords:** Evolution, Genetics

## Abstract

The semislug *Megaustenia siamensis*, commonly found in Thailand, is notable for its exceptional capacity to produce biological adhesives, enabling it to adhere to tree leaves even during heavy rainfall. In this study, we generated the first reference genome for *M. siamensis* using a combination of three sequencing technologies: Illumina’s short-read, Pac-Bio’s HIFI long-read, and Hi-C. The assembled genome size was 2593 billion base pairs (bp), containing 34,882 protein-coding genes. Our analysis revealed positive selection in pathways associated with the ubiquitin–proteasome system. Furthermore, RNA sequencing of foot and mantle tissues unveiled the primary constituents of the adhesive, including lectin-like proteins (C-lectin, H-lectin, and C1q) and matrilin-like proteins (VWA and EGF). Additionally, antimicrobial peptides were identified. The comprehensive *M. siamensis* genome and tissue-specific transcriptomic data provided here offer valuable resources for understanding its biology and exploring potential medical applications.

## Introduction

Terrestrial gastropods, fascinating invertebrates, originated from marine ancestors and evolved to diverse terrestrial habitats over time. Among these, pulmonates stand out; they possess a pallial lung for gas exchange instead of gills and are divided into three main groups: snails, semislugs, and slugs^1^. To thrive on land, these gastropods secrete slime or mucus with multiple functions, including adhesion, emollience, moisturization, lubrication, and defense^1,2^. This mucus contains various bioactive components, such as antibacterial and antioxidant compounds^3,4^. Consequently, there is a growing interest in exploring terrestrial gastropod mucus for medical, pharmaceutical, and cosmetic applications^5–8^. Studies have examined its protein components, glycosylation, ion content, and mechanical properties^2,4^, and advancements in genomic and transcriptomic data enable the reconstruction of snail mucus biosynthetic pathways^9^. However, most studies into the genomic structure and differential gene expression of terrestrial gastropods have focused predominantly on snails and slugs^10–12^, neglecting semislugs entirely.

*Megaustenia siamensis*, commonly known as the Siamese semislug, is abundant and inhabits various environments across Thailand. When disturbed, this creature secretes highly adhesive mucus, aiding in its firm attachment to surfaces. During the dry season, it can retract into its shell and secrete mucus to form an epiphragm over the shell aperture, protecting against desiccation^13,14^. Consequently, the mucus of the semislug exhibits distinctive properties compared to that of snails and slugs. Leveraging high-throughput sequencing technologies, we integrate genome and transcriptome data of *M. siamensis* to unveil its genetic characteristics, aiming to understand the properties of its mucus. This research is pivotal for advancing our understanding of semislug evolution and investigating potential medicinal applications of terrestrial gastropod mucus.

## Results

### *M. siamensis* genome assembly and annotation

**Figure 1:**
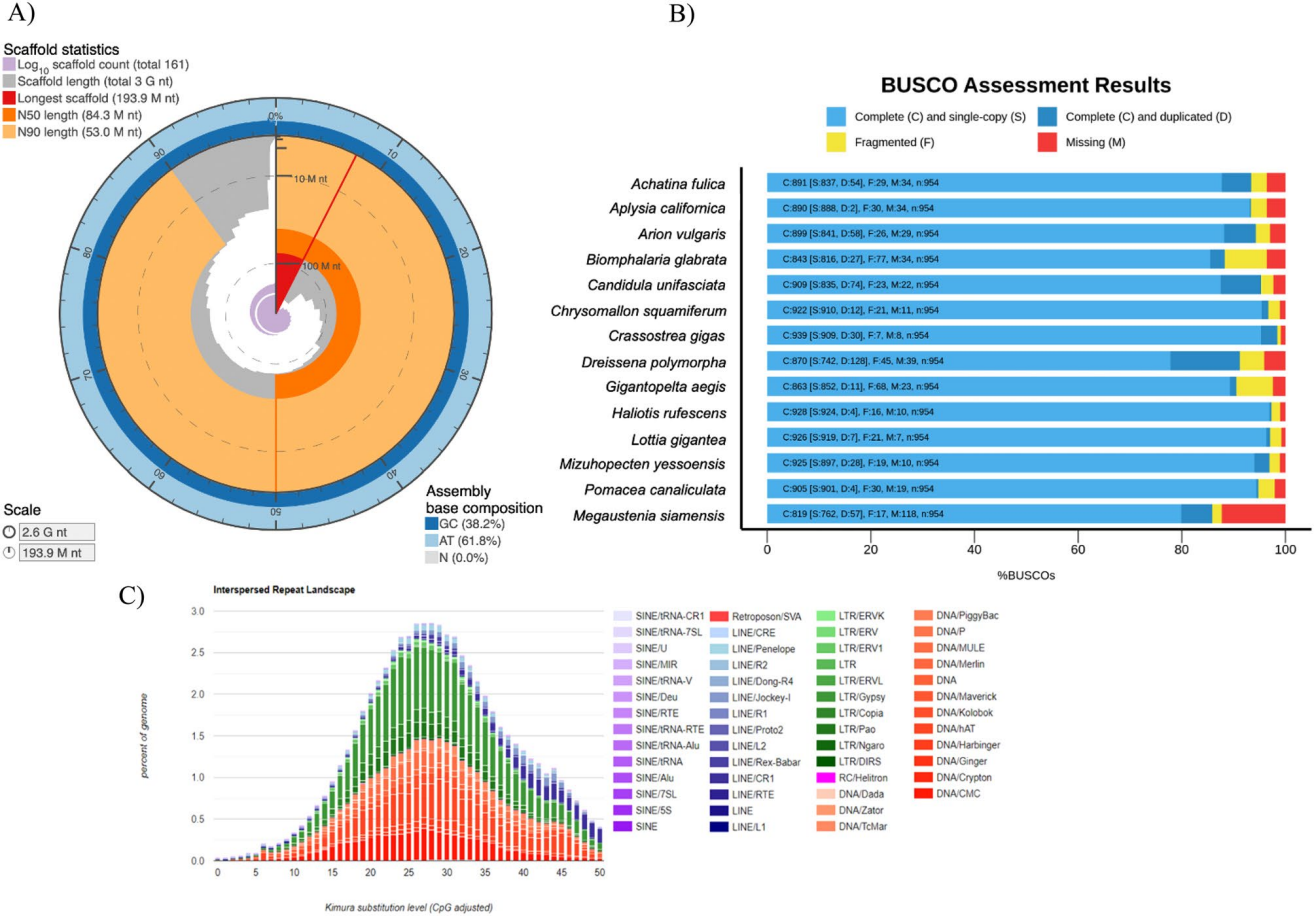
Summary statistics of *M. siamensis* genome (**A**). Bar chart with summary assessments for the proportion of genes present in snails. The summary assessment shows the percentage of complete and single copy genes (Light blue), complete and duplicated genes (Dark blue), fragmented genes (Yellow blue), and missing genes in the assemblies (Red blue) (**B**). Transposable element divergence landscape within the *M. siamensis* genome (different classes represented by different colors) (**C**).

The estimated genome size of *M. siamensis* is approximately 2.2 Gb, determined through *k*-mer analysis with short reads (Supplementary Fig. 1). We obtained 234 Gb of raw PacBio long reads, of which 196 Gb were filtered as clean reads. The initial draft assembly, generated using Canu software with default parameters, yielded an assembly of around 3.07 Gb, comprising 5246 contigs with an N50 contig length of 1.5 Mb. Subsequently, Illumina short reads were employed for polishing and error correction, resulting in a refined assembly of approximately 2.6 Gb, with an N50 of 1.8 Mb. The final draft genome assembly involved genomic scaffolding using Hi-C data. A total of 161 scaffolds were anchored into 32 pseudo-chromosomes (2n = 64), with a combined length of 2.59 Gb and an N50 of 84.3 Mb (Supplementary Fig. 2). The number of chromosome-scale scaffolds aligns with other terrestrial pulmonates, which typically range from 2n = 16 to 2n = 66 based on cytogenetic studies^15^. Genome assembly statistics are presented in Fig. 1A and Table 1. Evaluation of the genome completeness of *M. siamensis*, conducted by searching for 954 single-copy metazoan genes using Benchmarking Universal Single-Copy Orthologs (BUSCO), revealed a completeness level of 86.9%, indicating its adequacy as a genomic reference resource.

**Table 1:** Genome statistics of the *Megaustenia siamensis.*

Assembly	*Megaustenia siamensis*
Genome size (bp)	2593626580
Number of scaffolds	161
Scaffold N50	84301762
Number of protein-coding genes	34,882
Repeat content (%)	60.69
GC content (%)	38.24
Complete BUSCO (%)	85.9
Complete and Single-copy BUSCO (%)	79.9
Complete and Duplicated BUSCO (%)	6.0
Fragmented BUSCO (%)	1.8
Missing BUSCO (%)	12.3
Total number of metazoa_odb10	954

We further compared the proportion of genes present in 13 mollusk genomes (Supplementary Table 1 and Fig. 1B). The genome assembly size of the semislug *M. siamensis* is notably larger than that of other terrestrial snails and slugs (approximately 1.8 Gb for *Lissachatina fulica*, 1.5 Gb for *Arion vulgaris*, and 1.2 Gb for *Candidula unifasciata)*^10–12^. The GC content of *M. siamensis* is 38.24%, closely resembling that of *A. vulgaris* (38.46%). Repetitive elements constitute 60.69% of the genome (Fig. 1C and Supplementary Table 2). Annotation of the *M. siamensis* genome revealed a total of 34,882 protein-coding genes. Among these predicted protein-coding genes, approximately 58% could be annotated through at least one of the following protein-related databases: the EggNOG database (20,197; 57.90%), the Swiss-Prot protein database (14,181; 40.65%), the protein families (Pfam) database (18,863; 54.08%), and the Kyoto Encyclopedia of Genes and Genomes (KEGG) database (11,103; 31.83%).

### Gene family clustering

**Figure 2:**
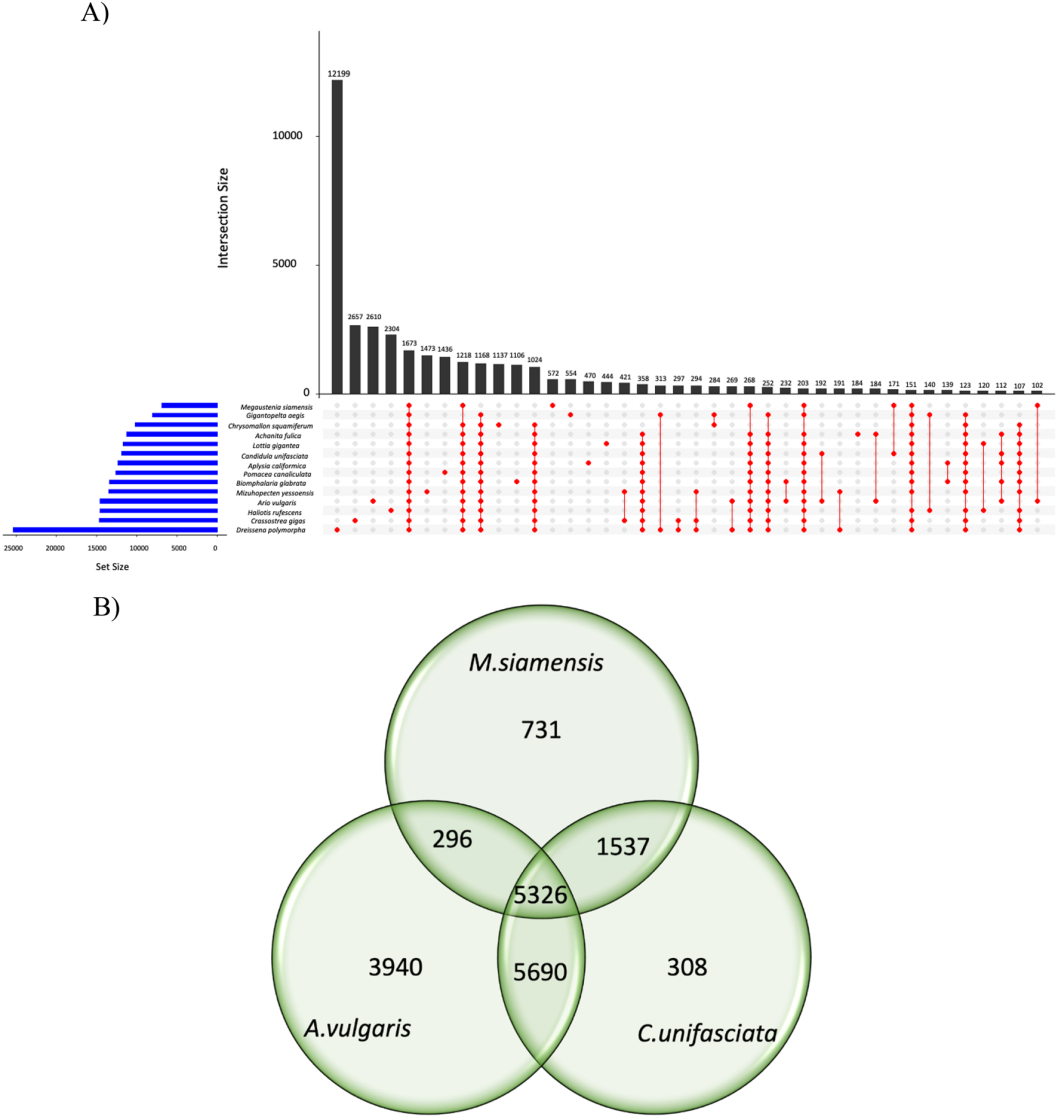
Orthologous gene cluster shared among the fourteen species (**A**). Venn diagram showing the distribution of gene families (orthologous clusters) among *M. siamensis*, *A. vulgaris*, and *C. unifasciata* (**B**).

A gene family is a set of genes with similar structures or functions, indicating adaptive evolution. Therefore, we investigate the adaptive evolution of *M. siamensis* by examining the relationship of gene families among 10 gastropod and 3 bivalve species. We identified 47,370 orthologous gene families containing a total of 668,284 genes. Among these, 1673 orthogroups were shared by all 14 species, with 26 of them represented as single-copy orthogroups (Fig. 2A). Illustrating the shared orthogroups among terrestrial snails and slugs, the Venn diagram revealed that 5326 gene families were shared among the slug *A. vulgaris*, the snail *C. unifasciata,* and *M. siamensis* (Fig. 2B). Out of these, 731 gene families were specific to *M. siamensis*, including 15 gene ontology (GO) enrichments (Supplementary Table 3). The top three significantly enriched gene families were related to the cellular component of the nucleus (GO: 0005634, *P* = 3.61e − 7, 8 genes), the biological process of xenobiotic metabolic process (GO: 0006805, *P* = 5.58e − 7, 7 genes), and transposition, DNA-mediated (GO: 0006313, *P* = 7.92e − 7, 5 genes). Additionally, two gene families concerning bacteria were significantly enriched: response to bacterium (GO: 0009617, *P* = 3.68e − 4, 4 genes), and negative regulation of defense response to bacterium, incompatible interaction (GO:1902478, *P* = 4.05e − 4, 2 genes), along with one concerning the regulation of innate immune response (GO:0045088, *P* = 1.32e − 3, 2 genes). Three significant GO enrichments were found between *M. siamensis* and *C. unifasciata* (Supplementary Table 4). Most proteins were significantly enriched in the gene families associated with the molecular function of G-protein-coupled receptor activity (GO: 0004930, *P* = 2.02e − 13, 20 genes), followed by the positive regulation of cytosolic calcium ion concentration (GO:0004930, *P* = 3.22e − 06, 11 genes), and outer dynein arm assembly (GO:0036158, *P* = 7.34e − 05, 8 genes). However, no significant GO enrichment was observed between *M. siamensis* and *A. vulgaris*.

### Phylogenetic construction and divergence time estimation

**Figure 3:**
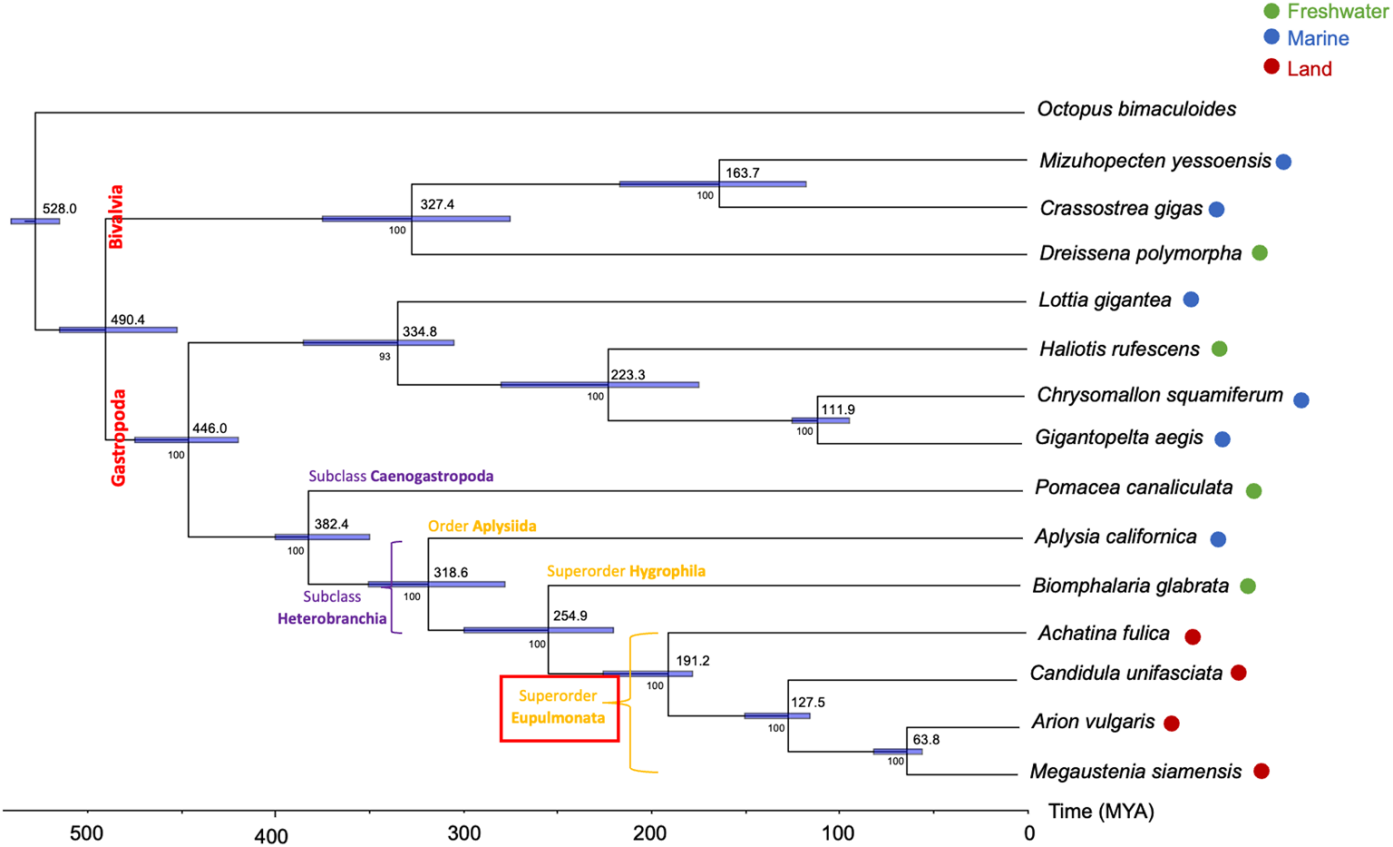
Phylogenetic relationship of *M. siamensis* with 4 freshwater, 6 marine, and 3 land snail. *Octopus bimaculoides* is outgroup. The divergence times (million year ago (MYA)) are shown with 95% confidence intervals represent blue color bar.

To understand the genomic evolution of *M. siamensis*, we analyzed a set of 26 single-copy orthologous genes shared among 10 gastropod and 3 bivalve species, with *Octopus bimaculoides* chosen as the outgroup species. The resulting phylogenetic tree revealed that *M. siamensis* is most closely related to *A. vulgaris*, with an estimated divergence time of approximately 63 million years ago (MYA). Within the remaining stylommatophoran snails, *C. unifasciata* emerged as a sister clade containing *M. siamensis* and *A. vulgaris*, while *L. fulica* forms a sister clade to these three stylommatophoran species. Furthermore, the analysis indicated the divergence of terrestrial pulmonates from other mollusks inhabiting different habitats, such as freshwater and marine snails, approximately 254 MYA. Additionally, the split between Gastropoda and Bivalvia occurred around 439 MYA (Fig. 3).

### Positive selection

Our further analysis exploring the roles of genes under positive selection (dn/ds > 1) revealed that 17 out of 26 orthologous groups exhibited evidence of positive selection in *M. siamensis* (Supplementary Table 5). The GO enrichment of genes under positive selection included 3 ontologies of biological processes, 7 cellular components, and 4 molecular functions (Supplementary Table 6). Additionally, we found evidence of pathway enrichment in the ubiquitin–proteasome pathway, specifically in the PSMD1 genes, with a raw *P*-value of 0.004. This finding supports the hypothesis of protein binding in *M. siamensis* through positive selection.

### Transcriptome assembly and functional annotation

The comparative transcriptome analysis of *M. siamensis* foot and mantle was conducted using the Illumina HiSeq sequencing platform. Approximately 30 Gb of data were assembled, resulting in 716,193 transcripts with an average length of 570 bp. The assembly comprised 403,122 unigenes, with a GC content of 39.19%. These unigenes were annotated in several databases, including Refseq (70,286, 17.44%), Pfam (69,212, 17.17%), clusters of orthologous groups (COG) (68,487, 16.99%), GO (64,830, 16.08%), and KEGG (5981, 1.48%). Notably, a total of 7830 genes were identified encoding putative antimicrobial peptides (AMPs) in the *M. siamensis* transcriptomes.

**Figure 4:**
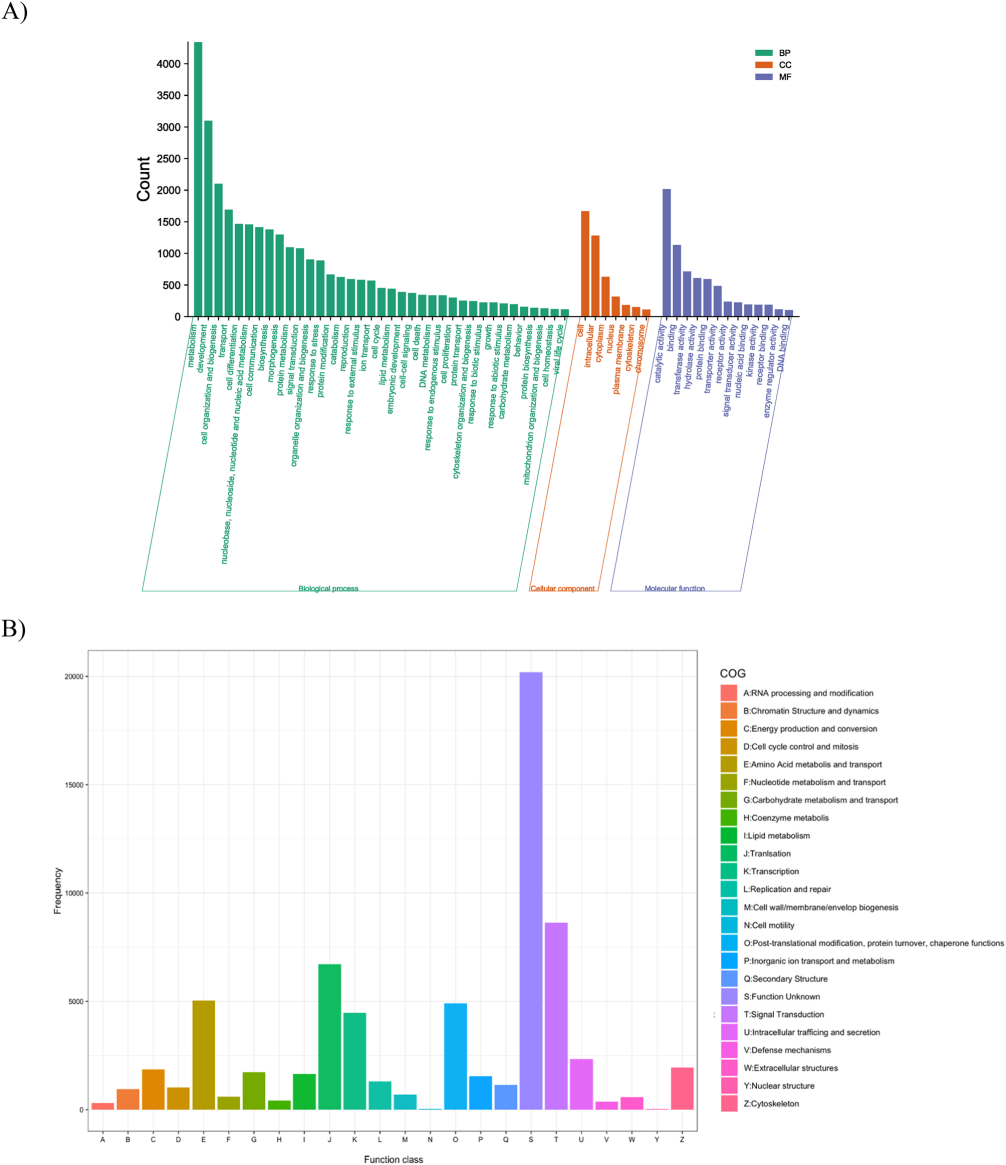
Function classification in Gene ontology (**A**) and Clusters of Orthologous Groups of proteins (COG) (**B**).

According to the GO annotation, a total of 64,830 unigenes were assigned to 113 GO terms covering biological processes, cellular components, and molecular functions. In the biological process category, the majority of unigenes were associated with metabolism (4342, 6.70%), development (3097, 4.77%), and cell organization and biogenesis (2102, 3.24%). Among the cellular component category, the most represented categories included cell (1667, 2.57%), intracellular (1281, 1.98%), and cytoplasm (630, 0.97%). In the molecular function category, the matched sequences were distributed across catalytic activity (2016, 3.11%), binding (1130, 1.74%), and transferase activity (715, 1.10%) (Fig. 4A).

Regarding COG annotation, a total of 68,487 unigenes were classified into 24 functional categories (Fig. 4B). The categories with the highest proportion of unigenes were unknown function (20,195, 29.49%), signal transduction mechanisms (8636, 12.61%), and translation, ribosomal structure, and biogenesis (6715, 9.80%). Additionally, 5981 unigenes were significantly matched in the KEGG database and assigned to 419 KEGG pathways. The metabolic pathways emerged as the pathway with the highest proportion of unigenes, followed by biosynthesis of secondary metabolites.

### Identification of differentially expressed genes

In the transcriptome analysis of *M. siamensis* foot and mantle, we obtained 390,055 assembled transcripts with an average length of 636 bp and identified 285,630 unigenes from the foot muscle data. Similarly, from the mantle data, we acquired 427,574 assembled transcripts with an average length of 555 bp and identified 255,705 unigenes. The GC percentage of the unigenes was 38.75% in the foot muscle and 39.32% in the mantle.

Differential gene expression analysis revealed a total of 115,263 differentially expressed genes in *M. siamensis* foot and mantle tissues. Among these, 55,675 genes were up-regulated, while 59,588 genes were down-regulated. The top 20 most highly expressed genes in the foot muscle and mantle are listed in Supplementary Tables 7 and 8, respectively. Notably, the top expressed genes in both tissues exhibit similarities. Particularly noteworthy is the higher expression of five genes involved in biological adhesion (actin, C1q, H-lectin, C-lectin, and VWA) in the foot muscle compared to the mantle, as detailed in Supplementary Tables 9 and 10.

### Antimicrobial and anticancer activity prediction

In the transcriptome of *M. siamensis*, a total of 7830 sequences were identified as putative AMPs. We conducted further analyses on the CAMP database for these sequences using four machine-learning algorithms, namely Support Vector Machine (SVM), Discriminant Analysis (DA), Artificial Neural Network (ANN), and Random Forest (RF). Additionally, the iACP tool was used for anticancer analysis.

**Figure 5:**
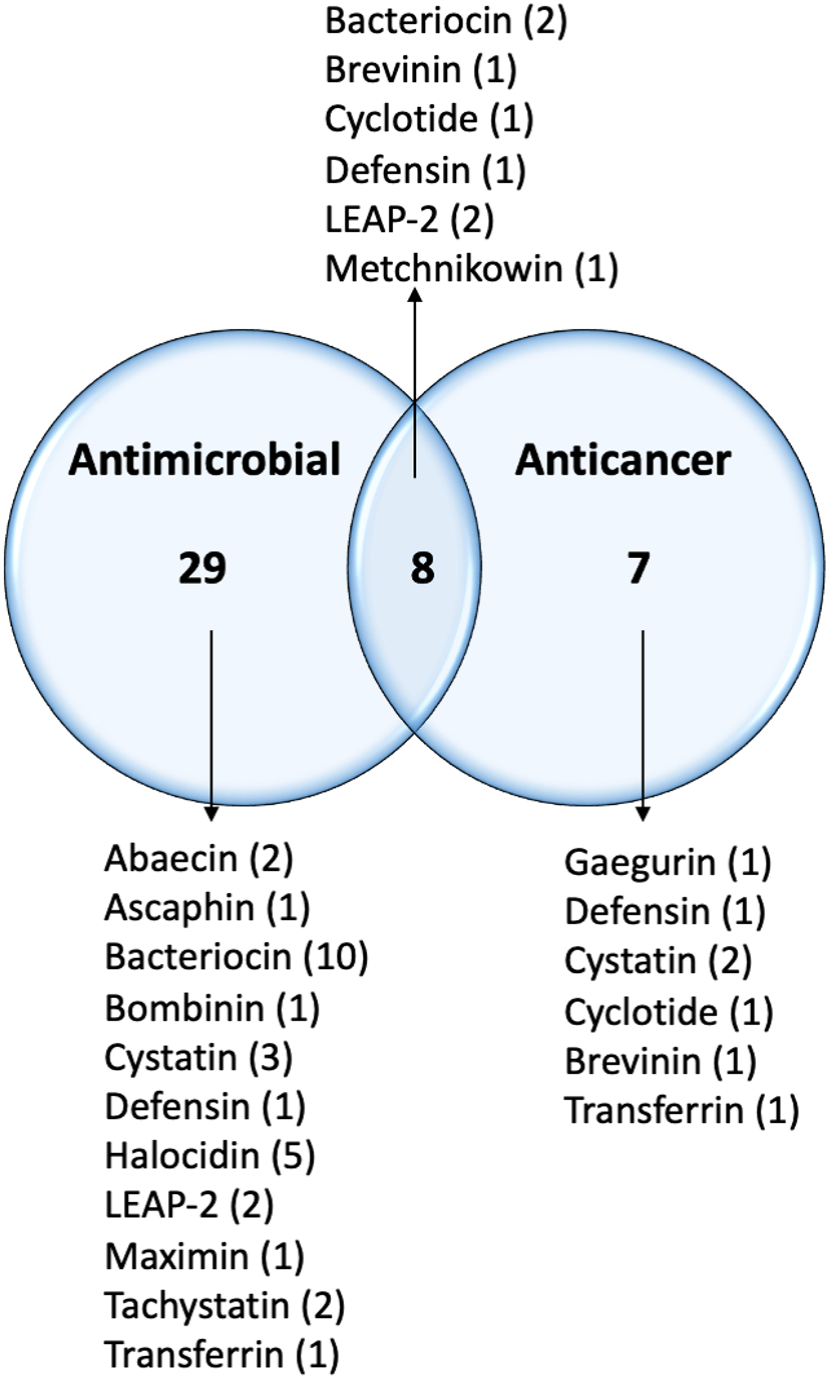
44 putative active peptides of antimicrobial and anticancer prediction.

The analysis yielded 44 putative active peptides. Among these, 29 sequences were predicted to possess antimicrobial properties, predominantly belonging to bacteriocin families. Eight sequences were identified to exhibit both putative antimicrobial and anticancer properties, while seven sequences were predicted to solely display anticancer activity (Supplementary Table 10 and Fig. 5). Furthermore, among these potential active peptides, eight peptides were found to be highly expressed in both foot (4 peptides) and mantle (4 peptides) tissues (Supplementary Table 10).

## Discussion

This study is the first comprehensive report on the de novo genome assembly of the semislug, a prominent member of the terrestrial pulmonates. Comparisons of gene proportions between *M. siamensis* and 13 other molluscan genomes (Supplementary Table 1 and Fig. 1B) indicate a higher prevalence of duplicated genes in terrestrial pulmonates. This elevated level of duplication may be attributed to a whole genome duplication event, similar to the one observed in the adaptation of giant African snails, *Lissachatina fulica*^11^.

Phylogenetic analysis revealed that *M. siamensis* emerges as a sister clade to *A. vulgaris* with strong support, aligning with previous molecular phylogenies based on nuclear ribosomal RNA^16,17^. However, the relationship between Limacoidei (which includes *M. siamensis*) and Arionoidea (to which *A. vulgaris* belongs) still exhibits lower support values. The clustering of *M. siamensis*, *A. vulgaris*, and *C. unifasciata* corresponds to their classification within the suborder Helicina (“nonachatinoid” clade), contrasting with *L. fulica* placed in the suborder Achatinina (“achatinoid” clade)^16–18^.

Among the significantly enriched gene families specific to *M. siamensis*, four gene ontologies (xenobiotic metabolic process, response to bacterium, negative regulation of defense response to bacterium, incompatible interaction, and regulation of innate immune response) emphasize the importance of the immune system and response to foreign substances in semislugs. This is further supported by the signature of positive selection observed in interleukin 1 receptor-associated kinase 1 binding protein 1 (IRAK1BP1), a major component of the Toll-like receptors signaling pathway, as seen in the putative immune signaling cascade of the land slug *Incilaria fruhstorferi*^19^. Moreover, our transcriptome analysis predicted the antimicrobial and anticancer properties of putative active peptides, consistent with findings from the mucus of other terrestrial gastropods. However, notable antimicrobial peptides such as achacin-like peptide, macin, and hemocyanin, identified in *L. fulica*, were not predicted in the transcriptome of *M. siamensis*^3,20–22^.

In contrast to transferrin and cystatin, which are prevalent across various animal taxa^23,24^ and widely reported from various documented in different molluscan groups^25–27^, reports of bacteriocin and defensin in mollusks are comparatively scarce. Molluscan homologs of bacteriocin have only been predicted from the whole genome of the zebra mussel, *Dreissena polymorpha*^28^, while molluscan defensins have been discovered in some species of the abalone genus, *Haliotis*^29–31^.

The remaining putative active peptides predicted in this study align with those reported from other animal groups. For example, abaecin from hymenopterans^32,33^, metchnikowin from the fruit fly genus *Drosophila*^34,35^, LEAP-2 from vertebrates^36^, brevinin and gaegurin from ranoid frogs^37–39^, and bombinin and maximin from the fire-bellied toad genus *Bombina*^40^. Some peptides predicted in this study have also been previously reported from a single species, such as halocidin from the tunicate *Halocynthia aurantium*^41^, tachystatin from the horseshoe crab *Tachypleus tridentatus*^42^, and ascaphin from the coastal tailed frog *Ascaphus truei*^43^.

The discovery of these putative active peptides from *M. siamensis* transcriptomes is novel, as they have not been reported in other mollusks before, particularly in terrestrial gastropods. This discovery offers valuable insights into their antimicrobial and anticancer potential, presenting opportunities for further research and development in the field of peptide-based therapeutics.

The analysis of differential gene expression revealed that five selected proteins involved in biological adhesion exhibited higher expression levels in the foot tissue compared to the mantle. These adhesion-related proteins fall into two major classes: lectin-like proteins (C-lectins, C1q, and H-lectins) and matrilin-like proteins (VWA and EGF). These proteins play crucial roles in cell adhesion and movement, aligning with the mechanism of glue-like mucus in terrestrial slugs^44,45^. Moreover, they also function in the molluscan innate immune system^46–49^. Notably, among the most highly expressed genes in the mantle transcriptome, the bovine pancreatic trypsin inhibitor BPTI/Kunitz domain is involved in defense against microbial pathogens^50^. These findings shed light on the molecular mechanisms underlying the biological adhesive and innate immunity-relevant properties of *M. siamensis* mucus, offering insights into the functional differences between foot and mantle tissues.

Overall, the genome and transcriptome data of *M. siamensis* not only enhance our understanding of the species itself but also hold broader implications for evolutionary biology, medical research, and the development of biotechnological applications. The draft genome of *M. siamensis* can serve as a valuable reference for future studies, aiding in the comprehension of evolutionary adaptations on land. Additionally, the transcriptome data provide insights into protein composition and bioactive compounds. The availability of this valuable resource is expected to catalyze further investigations and pave the way for future discoveries and advancements across various fields.

## Methods

### Sample collection and DNA sequencing

Specimens of *M. siamensis* were collected from Tham Sai Thong Temple, Kalasin, Thailand. Foot and mantle tissues were dissected from the snail’s body on dry ice to prevent RNA degradation. Genomic DNA extraction was done separately from the foot and mantle tissues using the DNAeasy^®^ Blood and Tissue kit from Qiagen™. DNA quantity and quality assessment were performed using a NanoDrop spectrophotometer (Thermo Scientific, Wilmington, DE) and an Agilent 2100 Bioanalyzer (Agilent Technologies).

We used three different sequencing technologies to obtain the genome sequence. Long-read DNA sequencing was conducted using the SMRTbell library prepared with the SMRTbell Express Template Prep Kit 2.0 (Pacific Biosciences) and sequenced on the PacBio Sequel sequencing system. A total of 234 Gb of data was generated from 33 SMRT cells, with an average insert length of 16,534 bp and an N50 read length of 27,177 bp.

For short-read DNA sequencing, approximately 200 Gb of data was produced using the TruSeq Nano DNA library prep kit and sequenced on the Illumina HiSeqX Ten platform (Illumina, San Diego, CA, USA) with 150 bp paired-end reads. Additionally, genomic DNA underwent Dovetail Omni-C library preparation using the Dovetail Hi-C preparation kit (Dovetail Genomics, Scotts Valley, CA, USA) following the manufacturer’s protocol (manual version 1.0 for non-mammalian samples). Sequencing was performed on the Illumina HiSeqX Ten platform with 150 bp paired-end reads.

### De novo genome assembly

Binary Alignment Map files containing the subreads generated by PacBio sequencing were converted to FASTA files using DEXTRACTOR software (https://github.com/thegenemyers/DEXTRACTOR). Subreads shorter than 1000 bp and those with quality scores lower than 0.80 were excluded. The resulting clean reads were used for de novo assembly using the CANU pipeline, involving three steps: correction, trimming, and assembly, with default parameters^51^. Subsequently, short reads were used for polishing by Pilon^52^, and redundant sequences in the assembly were eliminated using purge_dups^53^. Hi-C reads were then mapped to the de novo assembled contigs using BWA software to establish construct contacts among the contigs^54^. Finally, the HiRise scaffolding method was applied to connect the contigs together, resulting in the final assembly^55^.

### Genome assembly evaluation

The quality of the genome assemblies was assessed using descriptive measures, including the number of contigs, the total number of assembled bases, and completeness. This evaluation was conducted using the QUAST analysis tool^56^. Additionally, the BUSCO (version 5.2.2) was employed to evaluate the completeness of the genome. The metazoan version 10, comprising a set of 954 genes, served as the reference for this analysis. BUSCO was also run with identical parameters to facilitate comparison with 13 other species.

### Genome annotation

De novo repeat annotation was conducted using RepeatModeler (http://www.repeatmasker.org/ RepeatModeler/). The generated sequence libraries were subsequently utilized as queries to mask repetitive elements with RepeatMasker (http://www.repeatmasker.org). To calculate the Kimura divergence values, we employed the "calcDivergenceFromAlign.pl" script within the RepeatMasker pipeline. Additionally, the repeat landscape, encompassing the representation of repeats in the genomes, was visualized. For the annotation of protein-coding genes, we used the MAKER pipeline^57^.

Gene function annotation was conducted using InterProScan to search for domains or motifs in public databases. Additionally, we used the web-based platform KEGG^58,59^. Orthology assignments and predictions of KEGG pathways were performed through the KEGG Automatic Annotation Server using the bidirectional best hit (BBH) BLAST method (https://www.genome.jp/kegg/kaas/). Furthermore, the draft genomes underwent scanning for COGs annotations using eggNOG-mapper v2 (http://eggnog-mapper.embl.de).

### Gene family analysis

The protein sequences of *M. siamensis* were compared with those of 13 other species, including 10 gastropod species (*Achatina fulica*, *Aplysia californica*, *Arion vulgaris*, *Biomphalaria glabrata*, *Candidula unifasciata*, *Chrysomallon squamiferum* (Scaly-foot gastropod), *Gigantopelta aegis*, *Haliotis rufescens* (red abalone), *Lottia gigantea*, and *Pomacea canaliculate*) and 3 bivalvia species (*Crassostrea gigas* (Pacific oyster), *Dreissena polymorpha*, and *Mizuhopecten yessoensis*). OrthoFinder software (version 2.4.0)^60^ was used to identify gene family clusters among the different species, and the results were visualized using the UpSetR package^61^.

### Phylogenetic and divergence time analysis

A phylogenetic tree was constructed using one-to-one orthologous genes, identified through OrthoFinder analysis. The single-copy orthologous proteins were aligned using MUSCLE, and maximum likelihood trees were inferred using RAxML v8.2.8 with 1000 bootstrap replicates. Divergence times were computed using the MCMCTREE program implemented in the PAML v4.8 package, employing the correlated molecular clock^62^.

Four fossil calibration points were applied to estimate the divergence times: the divergence time of Gastropoda and Bivalvia (515.0–541.7 MYA), Caenogastropoda and Heterobranchia (238.6–429.9 MYA), *Pomacea canaliculata* and *Lottia gigantea* (238.6–429.9 MYA), and *Octopus bimaculoides* (480.0–559.4 MYA) for the root. These calibration pointpoints were obtained from the Timetree database (http://www.timetree.org/)^63^. The resulting phylogenetic tree was visualized using FigTree v.1.4.4 (http://tree.bio.ed.ac.uk/software/figtree/).

### Positive selection

To identify positive selection, one-to-one orthologous proteins were aligned using PRANK^64^, and codon alignments were generated using PAL2NAL^65^. The aBSREL model (adaptive Branch-Site Random Effects Likelihood) implemented in the Hyphy package (v2.5.15)^66^ was used to test whether positive selection had occurred on a proportion of branches (ω > 1), with a significance threshold of *P*-value of less than 0.05. Additionally, GO enrichment analysis was conducted using the PANTHER database.

### Transcriptome sequencing analysis

Ribonucleic acid was extracted separately from the foot and mantle tissues using the TruSeq Stranded mRNA LT Sample Prep Kit and sequenced on the Illumina Novaseq platform, resulting in 150 bp paired-end reads. The RNA-seq data underwent assembly using both de novo and genome-guided approaches with Trinity^67^. Redundant transcripts were identified and removed from the assembly using CD-HIT-EST^68^ with an identity threshold of 98% sequence similarity. We performed transcript quantification using RNA-Seq by Expectation–Maximization (RSEM)^69^. Normalization and differential expression analysis were conducted using EdgeR^70^. Candidate coding regions within transcript sequences were identified using the TransDecoder (https://github.com/TransDecoder/TransDecoder/wiki). Finally, functional annotation of the transcriptome was achieved using Trinotate, which involved conducting BLASTX searches on the Swiss-Prot databases to generate GO terms (http://trinotate.github.io).

The signal peptide was identified using SignalP 5.0^71^. Candidate peptides identified as antimicrobial were evaluated using four machine-learning algorithms: Support Vector Machine (SVM), DA, ANN, and Random Forest (RF), by mapping them with the Collection of Antimicrobial Peptides (CAMP)^72^. Additionally, the iACP online tool (https://lin.uestc.edu.cn/server/iACP) was utilized to predict the anticancer activity of the identified peptides.

### Supplementary Information

Supplementary Tables.

Supplementary Figures.

## Data Availability

The genome assembly generated in this study is available via NCBI under the BioProject number: PRJNA993791. All other relevant data are available upon request to the corresponding authors.
