# Supplementary material for: De novo genome assembly and transcriptome sequencing in foot and mantle tissues of Megaustenia siamensis reveals components of adhesive substances
Source: Sci Rep. 2024 Jun 14;14:13756. doi: 10.1038/s41598-024-64425-6 (PMC11178922; doi:10.1038/s41598-024-64425-6)
Supplement: Supplementary file 1 — Supplementary Tables. [file 41598_2024_64425_MOESM1_ESM.docx]

Supplementary Table 1. Genome statistics and sources used in this study (L: Land, F:Freshwater, M:Marine snails)

| **Habitat** | **Group** | **Species** | **Assembly size (Gb)** | **GC content (%)** | **Protein** | **Source** | **Accession** |
| --- | --- | --- | --- | --- | --- | --- | --- |
| L | Gastropoda | *Megaustenia siamensis* | 2.5 | 38.24 | 34882 | This study | This study |
| L | Gastropoda | *Achatina fulica* | 1.85 | NA | 23726 | GigaDB | DOI:10.5524/100647 |
| L | Gastropoda | *Arion vulgaris* | 1.54 | 38.46 | 32518 | NCBI | GCA_020796225.1 |
| L | Gastropoda | *Candidula unifasciata* | 1.36 | 40.3 | 22464 | NCBI | GCA_905116865.2 |
| F | Gastropoda | *Biomphalaria glabrata* | 0.92 | 36.1 | 36675 | NCBI | GCA_000457365.1 |
| F | Gastropoda | *Haliotis rufescens* | 1.33 | 40.9 | 55609 | NCBI | GCA_023055435.1 |
| F | Gastropoda | *Pomacea canaliculata* | 0.44 | 40.71 | 40391 | NCBI | GCA_003073045.1 |
| F | Bivalvia | *Dreissena polymorpha* | 1.8 | 35.13 | 189750 | NCBI | GCA_020536995.1 |
| M | Gastropoda | *Aplysia californica* | 0.93 | 41.99 | 26676 | NCBI | GCA_000002075.2 |
| M | Gastropoda | *Chrysomallon squamiferum* | 0.46 | 34.48 | 28781 | GigaDB | DOI:10.5524/100817 |
| M | Gastropoda | *Gigantopelta aegis* | 1.29 | 37.45 | 25601 | GigaDB | DOI:10.5524/100817 |
| M | Gastropoda | *Lottia gigantea* | 0.36 | 36 | 23822 | NCBI | GCA_000327385.1 |
| M | Bivalvia | *Crassostrea gigas* | 0.65 | 33.49 | 63341 | NCBI | GCA_902806645.1 |
| M | Bivalvia | *Mizuhopecten yessoensis* | 0.99 | 33.6 | 22448 | NCBI | GCA_002113885.2 |

Supplementary Table 2. Repetitive elements in *Megaustenia siamensis*

| sequences: 161 total length: 2,593,626,580 bp  GC level: 38.24 %  bases masked: 1,574,043,348 bp (60.69%) | | | | |
| --- | --- | --- | --- | --- |
| Type | SubType | No.of Element | Length (bp) | Proportion of Genome (%) |
| Retroelements | | 203642 | 37581094 | 1.45 |
| Penelope | | 6656 | 816445 | 0.03 |
| LINEs | | 98855 | 15964079 | 0.62 |
|  | L2/CR1/Rex | 18763 | 3793436 | 0.15 |
|  | R1/LOA/Jockey | 6106 | 1076663 | 0.04 |
|  | R2/R4/NeSL | 3529 | 531733 | 0.02 |
|  | RTE/Bov-B | 30438 | 4318208 | 0.17 |
|  | L1/CIN4 | 22840 | 3639015 | 0.14 |
| LTR elements: | | 102025 | 21488868 | 0.83 |
|  | BEL/Pao | 4955 | 950902 | 0.04 |
|  | Ty1/Copia | 17523 | 2960241 | 0.11 |
|  | Gypsy/DIRS1 | 51356 | 12954469 | 0.50 |
|  | Retroviral | 18317 | 1944650 | 0.07 |
| DNA transposons | | 232983 | 37035194 | 1.43 |
|  | hobo-Activator | 45873 | 8023241 | 0.31 |
|  | Tc1-IS630-Pogo | 19504 | 2768223 | 0.11 |
|  | PiggyBac | 1253 | 162483 | 0.01 |
|  | Tourist/Harbinger | 8048 | 1156016 | 0.04 |
| Rolling-circles | | 18023 | 3973667 | 0.15 |
| Unclassified: | | 4762262 | 1422041331 | 54.83 |
| Total interspersed repeats | | | 1496657619 | 57.71 |
| Small RNA | | 3061 | 196087 | 0.01 |
| Satellites | | 28442 | 14281899 | 0.55 |
| Simple repeats | | 534153 | 52875196 | 2.04 |
| Low complexity | | 56034 | 6135264 | 0.24 |

Supplementary Table 3. Function enrichment of specific gene families specific to *Megaustenia siamensis*

| GO ID | Category | Name | Count | p-value |
| --- | --- | --- | --- | --- |
| GO:0006805 | BP | xenobiotic metabolic process | 7 | 5.58E-07 |
| GO:0006313 | BP | transposition, DNA-mediated | 5 | 7.92E-07 |
| GO:0010951 | BP | negative regulation of endopeptidase activity | 3 | 6.24E-06 |
| GO:0009617 | BP | response to bacterium | 4 | 0.000367898 |
| GO:1902478 | BP | negative regulation of defense response to bacterium, incompatible interaction | 2 | 0.000405157 |
| GO:0006468 | BP | protein phosphorylation | 4 | 0.000663772 |
| GO:0045088 | BP | regulation of innate immune response | 2 | 0.001319505 |
| GO:0006310 | BP | DNA recombination | 2 | 0.002707308 |
| GO:0006493 | BP | protein O-linked glycosylation | 2 | 0.002707308 |
| GO:0003081 | BP | regulation of systemic arterial blood pressure by renin-angiotensin | 2 | 0.002707308 |
| GO:0032197 | BP | transposition, RNA-mediated | 2 | 0.005602581 |
| GO:0005634 | CC | nucleus | 8 | 3.61E-07 |
| GO:0003964 | MF | RNA-directed DNA polymerase activity | 4 | 5.51E-06 |
| GO:0004867 | MF | serine-type endopeptidase inhibitor activity | 2 | 0.003568037 |
| GO:0005344 | MF | oxygen transporter activity | 2 | 0.006768458 |

Supplementary Table 4. Function enrichment of specific gene families between *Megaustenia siamensis* and *Candidula unifasciata*

| GO ID | Category | Name | Count | p-value |
| --- | --- | --- | --- | --- |
| GO:0004930 | MF | G-protein coupled receptor activity | 20 | 2.0199E-13 |
| GO:0007204 | BP | positive regulation of cytosolic calcium ion concentration | 11 | 3.2246E-06 |
| GO:0036158 | BP | outer dynein arm assembly | 8 | 7.3392E-05 |

Supplementary Table 5. 17 orthologous groups with signatures of positive selection in *Megaustenia siamensis*

| Orthogroup | dn/ds | LRT | p-value | Gene |
| --- | --- | --- | --- | --- |
| OG0011944 | 8 | 30.813 | 6.68E-08 | Adenylate kinase 6 (AK6) |
| OG0012045 | 4.6 | 20.56 | 0.0000114 | Ribosomal protein L6 (RPL6) |
| OG0012047 | 10 | 145.49 | 0 | FtsJ RNA 2'-O-methyltransferase 3 (FTSJ3) |
| OG0012086 | 6.1 | 50.479 | 3.52E-12 | Flap structure-specific endonuclease 1 (FEN1) |
| OG0012116 | 4.9 | 100.65 | 0 | F-box and WD repeat domain containing 7 (FBXW7) |
| OG0012134 | 9.8 | 54.601 | 4.47E-13 | Sphingosine-1-phosphate lyase 1 (SGPL1) |
| OG0012140 | 5.8 | 85.577 | 0 | Signal recognition particle 68 (SRP68) |
| OG0012171 | 2.1 | 23.354 | 0.00000282 | Charged multivesicular body protein 3 (CHMP3) |
| OG0012254 | 10 | 24.968 | 0.00000125 | Proteasome assembly chaperone 2 (PSMG2) |
| OG0012257 | 13 | 52.642 | 1.19E-12 | Derlin 1 (DERL1) |
| OG0012313 | 19 | 63.727 | 4.66E-15 | Interleukin 1 receptor associated kinase 1 binding protein 1 (IRAK1BP1) |
| OG0012328 | 9.5 | 27.165 | 0.000000416 | - |
| OG0012417 | 5.8 | 27.467 | 0.000000358 | Proteasome 20S subunit beta 7 (PSMB7) |
| OG0012419 | 1.2 | 12.047 | 0.00083 | COP9 signalosome subunit 5 (COPS5) |
| OG0012425 | 3.9 | 23.954 | 0.00000208 | Proteasome 26S subunit, non-ATPase 1 (PSMD1) |
| OG0012429 | 6.6 | 79.786 | 0 | Structural maintenance of chromosomes 2 (SMC2) |
| OG0012431 | 7.2 | 101.56 | 0 | Nucleolar protein 10 (NOL10) |

Supplementary Table 6. Gene ontology enrichment of genes under positive selection

| Category | Term | Count | Genes | PValue |
| --- | --- | --- | --- | --- |
| BP | GO:0043161~proteasome-mediated ubiquitin-dependent protein catabolic process | 4 | PSMB7, FBXW7, DERL1, PSMD1 | 0.0000528 |
| BP | GO:0031648~protein destabilization | 2 | FBXW7, DERL1 | 0.03901364 |
| BP | GO:0001570~vasculogenesis | 2 | SGPL1, FBXW7 | 0.04798451 |
| CC | GO:0005730~nucleolus | 6 | FEN1, FBXW7, NOL10, SRP68, SMC2, FTSJ3 | 0.00232914 |
| CC | GO:0005829~cytosol | 10 | PSMB7, COPS5, FBXW7, CHMP3, PSMD1, PSMG2, SRP68, AK6, RPL6, SMC2 | 0.00682108 |
| CC | GO:0005634~nucleus | 10 | PSMB7, IRAK1BP1, FEN1, COPS5, FBXW7, PSMD1, PSMG2, AK6, RPL6, SMC2 | 0.01194283 |
| CC | GO:0005654~nucleoplasm | 8 | PSMB7, FEN1, COPS5, FBXW7, PSMD1, AK6, SMC2, FTSJ3 | 0.01462428 |
| CC | GO:0005694~chromosome | 3 | FBXW7, SMC2, FTSJ3 | 0.01469124 |
| CC | GO:0000502~proteasome complex | 2 | PSMB7, PSMD1 | 0.04097385 |
| CC | GO:0005783~endoplasmic reticulum | 4 | SGPL1, FBXW7, DERL1, SRP68 | 0.0476404 |
| MF | GO:0005047~signal recognition particle binding | 2 | DERL1, SRP68 | 0.00556939 |
| MF | GO:1990381~ubiquitin-specific protease binding | 2 | DERL1, CHMP3 | 0.01662143 |
| MF | GO:0005515~protein binding | 15 | FEN1, FBXW7, DERL1, SRP68, AK6, RPL6, SMC2, FTSJ3, PSMB7, IRAK1BP1, SGPL1, COPS5, CHMP3, PSMD1, PSMG2 | 0.01934424 |
| MF | GO:0031625~ubiquitin protein ligase binding | 3 | FBXW7, DERL1, PSMD1 | 0.02393807 |

Supplementary Table 7. The top 20 Most highly expressed genes in the Foot transcriptome.

| GeneID | PValue | FDR | Expected count | Description |
| --- | --- | --- | --- | --- |
| TRINITY_GG_26624_c14_g1 | 1.31E-55 | 4.89E-50 | 4920960.8 |  |
| TRINITY_GG_26465_c230_g1 | 1.63E-51 | 3.04E-46 | 1917161.26 | Actin |
| TRINITY_DN1068_c2_g1 | 5.09E-49 | 4.76E-44 | 1079062.74 |  |
| TRINITY_GG_58073_c6_g1 | 3.85E-48 | 2.88E-43 | 881383.73 |  |
| TRINITY_GG_2923_c17_g1 | 1.78E-47 | 1.11E-42 | 756239.52 |  |
| TRINITY_GG_23565_c7_g1 | 5.47E-47 | 2.92E-42 | 676045.46 |  |
| TRINITY_GG_3496_c104_g1 | 1.1E-46 | 4.55E-42 | 630522.71 |  |
| TRINITY_DN1068_c4_g1 | 2.9E-46 | 9.82E-42 | 572116.74 | VWA von Willebrand factor type A domain (COL6A3) |
| TRINITY_GG_30273_c12_g1 | 3.16E-46 | 9.82E-42 | 567335.7 |  |
| TRINITY_DN28462_c19_g1 | 3.82E-46 | 1.1E-41 | 556665.62 | Myosin tail |
| TRINITY_DN2111_c2_g1 | 5.98E-46 | 1.6E-41 | 532203.59 | VWA |
| TRINITY_GG_57043_c55_g1 | 8.29E-46 | 1.97E-41 | 515122.85 | Tropomyosin |
| TRINITY_DN747_c1_g2 | 5.07E-45 | 9.97E-41 | 429752.04 | GTP_EFTU Elongation factor Tu GTP binding domain (eef1a) |
| TRINITY_GG_17698_c0_g1 | 1.27E-44 | 2.03E-40 | 392139.84 | Lectin_C Lectin C-type domain (CLEC4E) |
| TRINITY_GG_40117_c18_g1 | 1.3E-44 | 2.03E-40 | 391077.26 |  |
| TRINITY_GG_2044_c58_g1 | 1.36E-44 | 2.03E-40 | 389519.44 | Myosin_tail_1 |
| TRINITY_GG_28374_c55_g1 | 1.36E-44 | 2.03E-40 | 389360.14 | EF-hand_6 |
| TRINITY_GG_8818_c120_g1 | 2.74E-44 | 3.42E-40 | 362997.33 |  |
| TRINITY_GG_49898_c29_g1 | 6.31E-44 | 6.93E-40 | 333985.07 | VWA (COL6A6) |

Supplementary Table 8. The top 20 Most highly expressed genes in the Mantle transcriptome.

| GeneID | PValue | FDR | Expected count | Description |
| --- | --- | --- | --- | --- |
| TRINITY_GG_27001_c17_g1 | 7.61E-50 | 9.47E-45 | 1184032.67 |  |
| TRINITY_GG_2568_c1348_g1 | 8.05E-47 | 3.76E-42 | 590117.22 |  |
| TRINITY_DN1503_c55_g1 | 2.03E-46 | 7.59E-42 | 537900.98 |  |
| TRINITY_DN1830_c0_g1 | 1.11E-43 | 1.15E-39 | 504381.52 |  |
| TRINITY_GG_1231_c6_g1 | 8.43E-46 | 1.97E-41 | 466578.89 |  |
| TRINITY_GG_2568_c1_g1 | 2.11E-45 | 4.63E-41 | 425713.02 |  |
| TRINITY_GG_11003_c29_g1 | 2.33E-45 | 4.83E-41 | 421511.74 |  |
| TRINITY_GG_2568_c9_g1 | 6.05E-45 | 1.13E-40 | 383078.98 |  |
| TRINITY_GG_25287_c221_g1 | 1.32E-44 | 2.03E-40 | 354402.4 | Actin |
| TRINITY_GG_8829_c14_g1 | 1.85E-44 | 2.66E-40 | 342572.65 |  |
| TRINITY_DN610_c0_g2 | 1.96E-44 | 2.71E-40 | 340640 | Kunitz_BPTI^Kunitz/Bovine pancreatic trypsin inhibitor domain |
| TRINITY_GG_2568_c18_g1 | 2.13E-44 | 2.84E-40 | 337791.24 | Chitin binding Peritrophin-A domain |
| TRINITY_GG_24988_c45_g1 | 2.49E-44 | 3.2E-40 | 332583.65 |  |
| TRINITY_GG_45343_c185_g1 | 2.92E-44 | 3.52E-40 | 327313.8 |  |
| TRINITY_DN2216_c0_g2 | 3.8E-44 | 4.43E-40 | 318838.31 | VWA von Willebrand factor type A domain (Col6A6) |
| TRINITY_GG_25019_c3_g1 | 4.34E-44 | 4.92E-40 | 314564.03 |  |
| TRINITY_GG_47492_c21_g1 | 7.12E-44 | 7.6E-40 | 299396.41 |  |
| TRINITY_DN1_c45_g2 | 3.47E-43 | 3.09E-39 | 255510.02 | GTP_EFTU Elongation factor Tu GTP binding domain (eef1a) |
| TRINITY_GG_8506_c29_g2 | 4.42E-43 | 3.75E-39 | 249444.38 |  |
| TRINITY_DN3375_c0_g1 | 5.88E-27 | 1.32E-24 | 247746.27 | HMG_box_2 (ND2) |

Supplementary Table 9. Number of gene considered as expressed, involving the glue mechanism, in foot tissue and localization mapped onto the genome assembly.

| Foot-transcriptID | Genes | LogCPM | PValue | FDR | Accession | Start-Stop |
| --- | --- | --- | --- | --- | --- | --- |
| TRINITY_GG_26465_c230_g1 | Actin | 13.8849995 | 1.63E-51 | 3.04E-46 | [JAVIAB010000159.1](https://www.ncbi.nlm.nih.gov/nucleotide/JAVIAB010000159.1?report=genbank&log$=nucltop&blast_rank=1&RID=0N0WB18Y013) | 18918959 - 1891990 |
| TRINITY_GG_18535_c0_g1 | Actin | 9.18056359 | 2.36E-37 | 4.66E-34 | [JAVIAB010000160.1](https://www.ncbi.nlm.nih.gov/nucleotide/JAVIAB010000160.1?report=genbank&log$=nucltop&blast_rank=1&RID=0N0WB18Y013) | 25720573 - 25721405 |
| TRINITY_GG_18535_c0_g2 | Actin | 8.73606759 | 5.13E-36 | 7.48E-33 | [JAVIAB010000160.1](https://www.ncbi.nlm.nih.gov/nucleotide/JAVIAB010000160.1?report=genbank&log$=nucltop&blast_rank=1&RID=0N0WB18Y013) | 25714386 - 25721405 |
| TRINITY_GG_8413_c76_g1 | C1q | 11.0117623 | 7.25E-43 | 5.76E-39 | [JAVIAB010000108.1](https://www.ncbi.nlm.nih.gov/nucleotide/JAVIAB010000108.1?report=genbank&log$=nucltop&blast_rank=1&RID=0NDU7RXF01N) | 52156607 - 52156839 |
| TRINITY_GG_28714_c72_g1 | C1q | 10.6097577 | 1.18E-41 | 6.36E-38 | [JAVIAB010000108.1](https://www.ncbi.nlm.nih.gov/nucleotide/JAVIAB010000108.1?report=genbank&log$=nucltop&blast_rank=1&RID=0NDU7RXF01N) | 46596008 - 46596242 |
| TRINITY_GG_10165_c0_g1 | C1q | 10.5483946 | 1.80E-41 | 9.20E-38 | [JAVIAB010000108.1](https://www.ncbi.nlm.nih.gov/nucleotide/JAVIAB010000108.1?report=genbank&log$=nucltop&blast_rank=1&RID=0NDU7RXF01N) | 52166521 - 52166647 |
| TRINITY_GG_9343_c0_g1 | C1q | 9.93586843 | 1.26E-39 | 4.46E-36 | [JAVIAB010000145.1](https://www.ncbi.nlm.nih.gov/nucleotide/JAVIAB010000145.1?report=genbank&log$=nucltop&blast_rank=1&RID=0NDU7RXF01N) | 47149773 - 47150045 |
| TRINITY_GG_28714_c17_g1 | C1q | 9.85350146 | 2.22E-39 | 7.54E-36 | [JAVIAB010000108.1](https://www.ncbi.nlm.nih.gov/nucleotide/JAVIAB010000108.1?report=genbank&log$=nucltop&blast_rank=1&RID=0NDU7RXF01N) | 52156663 - 52156839 |
| TRINITY_GG_28682_c30_g1 | C1q | 9.73711869 | 4.98E-39 | 1.51E-35 | [JAVIAB010000108.1](https://www.ncbi.nlm.nih.gov/nucleotide/JAVIAB010000108.1?report=genbank&log$=nucltop&blast_rank=1&RID=0NDU7RXF01N) | 45557994 - 45558247 |
| TRINITY_GG_26465_c9_g1 | C1q | 9.23006805 | 1.67E-37 | 3.41E-34 | [JAVIAB010000108.1](https://www.ncbi.nlm.nih.gov/nucleotide/JAVIAB010000108.1?report=genbank&log$=nucltop&blast_rank=1&RID=0NDU7RXF01N) | 51816915 - 51817127 |
| TRINITY_GG_34600_c46_g1 | C1q | 8.27214588 | 1.28E-34 | 1.36E-31 | [JAVIAB010000101.1](https://www.ncbi.nlm.nih.gov/nucleotide/JAVIAB010000101.1?report=genbank&log$=nucltop&blast_rank=1&RID=0NDU7RXF01N) | 1561976 - 1562936 |
| TRINITY_GG_8818_c11_g1 | C1q | 7.98735532 | 9.19E-34 | 8.19E-31 | [JAVIAB010000108.1](https://www.ncbi.nlm.nih.gov/nucleotide/JAVIAB010000108.1?report=genbank&log$=nucltop&blast_rank=1&RID=0NDU7RXF01N) | 51816981 - 51817127 |
| TRINITY_DN20231_c0_g1 | C1q | 7.90208652 | 7.96E-16 | 2.05E-14 | [JAVIAB010000108.1](https://www.ncbi.nlm.nih.gov/nucleotide/JAVIAB010000108.1?report=genbank&log$=nucltop&blast_rank=1&RID=0NDU7RXF01N) | 46643066 - 46643303 |
| TRINITY_DN32375_c0_g1 | C1q | 7.61479371 | 1.34E-31 | 7.34E-29 | [JAVIAB010000108.1](https://www.ncbi.nlm.nih.gov/nucleotide/JAVIAB010000108.1?report=genbank&log$=nucltop&blast_rank=1&RID=0NDU7RXF01N) | 46623496 - 46623705 |
| TRINITY_DN1414_c4_g1 | C1q | 7.26005501 | 1.42E-28 | 4.24E-26 | [JAVIAB010000108.1](https://www.ncbi.nlm.nih.gov/nucleotide/JAVIAB010000108.1?report=genbank&log$=nucltop&blast_rank=1&RID=0NDU7RXF01N) | 48448724 - 48449463 |
| TRINITY_DN857_c24_g1 | H_lectin | 11.0309161 | 6.35E-43 | 5.15E-39 | [JAVIAB010000145.1](https://www.ncbi.nlm.nih.gov/nucleotide/JAVIAB010000145.1?report=genbank&log$=nucltop&blast_rank=1&RID=0NDUGWBA01N) | 38601009 - 38601464 |
| TRINITY_GG_49449_c30_g1 | H_lectin | 7.63560423 | 1.05E-32 | 7.36E-30 | [JAVIAB010000124.1](https://www.ncbi.nlm.nih.gov/nucleotide/JAVIAB010000124.1?report=genbank&log$=nucltop&blast_rank=1&RID=0NDUGWBA01N) | 40693969 - 40694683 |
| TRINITY_GG_17698_c0_g1 | Lectin_C | 11.595487 | 1.27E-44 | 2.03E-40 | [JAVIAB010000059.1](https://www.ncbi.nlm.nih.gov/nucleotide/JAVIAB010000059.1?report=genbank&log$=nucltop&blast_rank=1&RID=0NEYBK0E015) | 62877805 - 62878112 |
| TRINITY_GG_17677_c0_g1 | Lectin_C | 9.92221666 | 1.38E-39 | 4.86E-36 | [JAVIAB010000059.1](https://www.ncbi.nlm.nih.gov/nucleotide/JAVIAB010000059.1?report=genbank&log$=nucltop&blast_rank=1&RID=0NEYBK0E015) | 62561565 - 62561723 |
| TRINITY_DN1283_c1_g1 | Lectin_C | 9.75537171 | 4.39E-39 | 1.34E-35 | [JAVIAB010000059.1](https://www.ncbi.nlm.nih.gov/nucleotide/JAVIAB010000059.1?report=genbank&log$=nucltop&blast_rank=1&RID=0NEYBK0E015) | 61549971 - 61550139 |
| TRINITY_GG_10290_c23_g1 | Lectin_C | 8.61242683 | 1.21E-35 | 1.65E-32 | [JAVIAB010000091.1](https://www.ncbi.nlm.nih.gov/nucleotide/JAVIAB010000091.1?report=genbank&log$=nucltop&blast_rank=1&RID=0NEYBK0E015) | 9228157 - 9228515 |
| TRINITY_GG_16364_c20_g1 | Lectin_C | 7.93639953 | 1.31E-33 | 1.12E-30 | [JAVIAB010000059.1](https://www.ncbi.nlm.nih.gov/nucleotide/JAVIAB010000059.1?report=genbank&log$=nucltop&blast_rank=1&RID=0NEYBK0E015) | 61549974 - 61550139 |
| TRINITY_DN42074_c1_g1 | Lectin_C | 7.32382726 | 2.61E-29 | 8.92E-27 | [JAVIAB010000059.1](https://www.ncbi.nlm.nih.gov/nucleotide/JAVIAB010000059.1?report=genbank&log$=nucltop&blast_rank=1&RID=0NEYBK0E015) | 62526733 - 62526927 |
| TRINITY_DN1068_c4_g1 | VWA | 12.1404211 | 2.90E-46 | 9.82E-42 | [JAVIAB010000088.1](https://www.ncbi.nlm.nih.gov/nucleotide/JAVIAB010000088.1?report=genbank&log$=nucltop&blast_rank=1&RID=0NEWBJH8015) | 14156569 - 1415683 |
| TRINITY_DN2111_c2_g1 | VWA | 12.0360915 | 5.98E-46 | 1.60E-41 | [JAVIAB010000046.1](https://www.ncbi.nlm.nih.gov/nucleotide/JAVIAB010000046.1?report=genbank&log$=nucltop&blast_rank=1&RID=0NEWBJH8015) | 8644683 - 8645108 |
| TRINITY_GG_49898_c29_g1 | VWA | 11.3639055 | 6.31E-44 | 6.93E-40 | [JAVIAB010000007.1](https://www.ncbi.nlm.nih.gov/nucleotide/JAVIAB010000007.1?report=genbank&log$=nucltop&blast_rank=1&RID=0NEWBJH8015) | 231341 - 231587 |
| TRINITY_GG_8413_c1_g1 | VWA | 11.1653386 | 2.50E-43 | 2.28E-39 | [JAVIAB010000088.1](https://www.ncbi.nlm.nih.gov/nucleotide/JAVIAB010000088.1?report=genbank&log$=nucltop&blast_rank=1&RID=0NEWBJH8015) | 13788341 - 13788610 |
| TRINITY_DN1717_c3_g1 | VWA | 9.30323497 | 1.01E-37 | 2.24E-34 | [JAVIAB010000046.1](https://www.ncbi.nlm.nih.gov/nucleotide/JAVIAB010000046.1?report=genbank&log$=nucltop&blast_rank=1&RID=0NEWBJH8015) | 53376475 - 53377793 |
| TRINITY_GG_8413_c0_g1 | VWA | 9.14836426 | 2.95E-37 | 5.61E-34 | [JAVIAB010000088.1](https://www.ncbi.nlm.nih.gov/nucleotide/JAVIAB010000088.1?report=genbank&log$=nucltop&blast_rank=1&RID=0NEWBJH8015) | 14198188 - 14198444 |
| TRINITY_DN110906_c2_g1 | VWA | 9.09160918 | 4.36E-37 | 8.03E-34 | [JAVIAB010000046.1](https://www.ncbi.nlm.nih.gov/nucleotide/JAVIAB010000046.1?report=genbank&log$=nucltop&blast_rank=1&RID=0NEWBJH8015) | 9534600 - 9534829 |
| TRINITY_DN1279_c2_g2 | VWA | 9.01536221 | 7.40E-37 | 1.30E-33 | [JAVIAB010000046.1](https://www.ncbi.nlm.nih.gov/nucleotide/JAVIAB010000046.1?report=genbank&log$=nucltop&blast_rank=1&RID=0NEWBJH8015) | 8495921 - 8496151 |
| TRINITY_DN1007_c0_g2 | VWA | 8.279822 | 1.21E-34 | 1.30E-31 | [JAVIAB010000145.1](https://www.ncbi.nlm.nih.gov/nucleotide/JAVIAB010000145.1?report=genbank&log$=nucltop&blast_rank=1&RID=0NEWBJH8015) | 34700024 - 34702177 |
| TRINITY_GG_14650_c51_g1 | VWA | 8.24707184 | 1.52E-34 | 1.58E-31 | [JAVIAB010000008.1](https://www.ncbi.nlm.nih.gov/nucleotide/JAVIAB010000008.1?report=genbank&log$=nucltop&blast_rank=1&RID=0NEWBJH8015) | 2065959 - 2066431 |
| TRINITY_GG_46066_c1_g1 | VWA | 8.03587871 | 6.57E-34 | 6.00E-31 | [JAVIAB010000046.1](https://www.ncbi.nlm.nih.gov/nucleotide/JAVIAB010000046.1?report=genbank&log$=nucltop&blast_rank=1&RID=0NEWBJH8015) | 8434153 - 8434378 |
| TRINITY_DN48862_c0_g3 | VWA | 7.57933263 | 1.55E-32 | 1.06E-29 | [JAVIAB010000046.1](https://www.ncbi.nlm.nih.gov/nucleotide/JAVIAB010000046.1?report=genbank&log$=nucltop&blast_rank=1&RID=0NEWBJH8015) | 13549797 - 13550020 |
| TRINITY_GG_10165_c49_g1 | VWA | 7.5536883 | 1.86E-32 | 1.24E-29 | [JAVIAB010000046.1](https://www.ncbi.nlm.nih.gov/nucleotide/JAVIAB010000046.1?report=genbank&log$=nucltop&blast_rank=1&RID=0NEWBJH8015) | 13549797 - 13550020 |
| TRINITY_DN26479_c0_g1 | VWA | 7.48929806 | 5.62E-28 | 1.53E-25 | [JAVIAB010000007.1](https://www.ncbi.nlm.nih.gov/nucleotide/JAVIAB010000007.1?report=genbank&log$=nucltop&blast_rank=1&RID=0NEWBJH8015) | 25637 - 25878 |
| TRINITY_DN41330_c0_g1 | VWA | 7.21636671 | 1.92E-31 | 1.03E-28 | [JAVIAB010000046.1](https://www.ncbi.nlm.nih.gov/nucleotide/JAVIAB010000046.1?report=genbank&log$=nucltop&blast_rank=1&RID=0NEWBJH8015) | 14680009 - 14680239 |

Supplementary Table 10. Number of gene considered as expressed, involving the glue mechanism, in mantle tissue and localization mapped onto the genome assembly.

| Mantle-transcriptID | Genes | LogCPM | PValue | FDR | Accession | Start-Stop |
| --- | --- | --- | --- | --- | --- | --- |
| TRINITY_GG_25287_c221_g1 | Actin | 11.5898368 | 1.32E-44 | 2.03E-40 | JAVIAB010000159.1 | 18918959 - 18919909 |
| TRINITY_GG_18863_c0_g1 | Actin | 10.311264 | 7.45E-37 | 1.30E-33 | [JAVIAB010000160.1](https://www.ncbi.nlm.nih.gov/nucleotide/JAVIAB010000160.1?report=genbank&log$=nucltop&blast_rank=1&RID=0NCME7PF016) | 25686978 - 25688301 |
| TRINITY_DN4875_c0_g3 | C1q | 10.5329776 | 2.00E-41 | 9.96E-38 | [JAVIAB010000108.1](https://www.ncbi.nlm.nih.gov/nucleotide/JAVIAB010000108.1?report=genbank&log$=nucltop&blast_rank=1&RID=0N2JK51N013) | 46596008 - 46596242 |
| TRINITY_GG_26467_c0_g1 | C1q | 10.1309401 | 3.25E-40 | 1.32E-36 | [JAVIAB010000108.1](https://www.ncbi.nlm.nih.gov/nucleotide/JAVIAB010000108.1?report=genbank&log$=nucltop&blast_rank=1&RID=0N2JK51N013) | 46643066 - 46643303 |
| TRINITY_GG_2568_c125_g1 | C1q | 9.41944499 | 4.50E-38 | 1.11E-34 | [JAVIAB010000145.1](https://www.ncbi.nlm.nih.gov/nucleotide/JAVIAB010000145.1?report=genbank&log$=nucltop&blast_rank=1&RID=0N2JK51N013) | 47149773 - 47150019 |
| TRINITY_GG_55553_c51_g1 | C1q | 8.95091616 | 1.16E-36 | 1.95E-33 | [JAVIAB010000145.1](https://www.ncbi.nlm.nih.gov/nucleotide/JAVIAB010000145.1?report=genbank&log$=nucltop&blast_rank=1&RID=0N2JK51N013) | 47132593 - 47132730 |
| TRINITY_GG_8829_c0_g1 | C1q | 8.01452835 | 7.61E-34 | 6.83E-31 | [JAVIAB010000145.1](https://www.ncbi.nlm.nih.gov/nucleotide/JAVIAB010000145.1?report=genbank&log$=nucltop&blast_rank=1&RID=0N2JK51N013) | 47146362 - 47146582 |
| TRINITY_GG_18863_c28_g1 | C1q | 7.76691884 | 4.23E-33 | 3.24E-30 | [JAVIAB010000108.1](https://www.ncbi.nlm.nih.gov/nucleotide/JAVIAB010000108.1?report=genbank&log$=nucltop&blast_rank=1&RID=0N2JK51N013) | 51977641 - 51977767 |
| TRINITY_DN1663_c0_g1 | C1q | 7.74976506 | 4.76E-33 | 3.62E-30 | [JAVIAB010000108.1](https://www.ncbi.nlm.nih.gov/nucleotide/JAVIAB010000108.1?report=genbank&log$=nucltop&blast_rank=1&RID=0N2JK51N013) | 52156612 - 52156839 |
| TRINITY_GG_55553_c56_g1 | C1q | 7.60214034 | 1.32E-32 | 9.08E-30 | [JAVIAB010000108.1](https://www.ncbi.nlm.nih.gov/nucleotide/JAVIAB010000108.1?report=genbank&log$=nucltop&blast_rank=1&RID=0N2JK51N013) | 46628206 - 46628343 |
| TRINITY_GG_14603_c40_g1 | C1q | 7.43593158 | 4.19E-32 | 2.56E-29 | [JAVIAB010000008.1](https://www.ncbi.nlm.nih.gov/nucleotide/JAVIAB010000008.1?report=genbank&log$=nucltop&blast_rank=1&RID=0N2JK51N013) | 54473260 - 54473481 |
| TRINITY_GG_39734_c91_g1 | C1q | 7.15963275 | 2.84E-31 | 1.46E-28 | [JAVIAB010000108.1](https://www.ncbi.nlm.nih.gov/nucleotide/JAVIAB010000108.1?report=genbank&log$=nucltop&blast_rank=1&RID=0N2JK51N013) | 52156678 - 52156839 |
| TRINITY_GG_28651_c91_g1 | H_lectin | 9.42228248 | 4.41E-38 | 1.10E-34 | [JAVIAB010000145.1](https://www.ncbi.nlm.nih.gov/nucleotide/JAVIAB010000145.1?report=genbank&log$=nucltop&blast_rank=1&RID=0NCTSZ9401N) | 38601132 - 38601464 |
| TRINITY_DN1240_c1_g1 | H_lectin | 7.82545541 | 2.82E-33 | 2.24E-30 | [JAVIAB010000008.1](https://www.ncbi.nlm.nih.gov/nucleotide/JAVIAB010000008.1?report=genbank&log$=nucltop&blast_rank=1&RID=0NCTSZ9401N) | 40029890 - 40030865 |
| TRINITY_GG_16167_c0_g1 | Lectin_C | 10.9852856 | 8.71E-43 | 6.77E-39 | [JAVIAB010000059.1](https://www.ncbi.nlm.nih.gov/nucleotide/JAVIAB010000059.1?report=genbank&log$=nucltop&blast_rank=1&RID=0NCUGZ5801N) | 62877717 - 62878112 |
| TRINITY_DN8905_c0_g1 | Lectin_C | 9.51920915 | 2.25E-38 | 5.97E-35 | [JAVIAB010000059.1](https://www.ncbi.nlm.nih.gov/nucleotide/JAVIAB010000059.1?report=genbank&log$=nucltop&blast_rank=1&RID=0NCUGZ5801N) | 62945125 - 62945502 |
| TRINITY_GG_2568_c76_g1 | Lectin_C | 9.35086494 | 7.23E-38 | 1.67E-34 | [JAVIAB010000059.1](https://www.ncbi.nlm.nih.gov/nucleotide/JAVIAB010000059.1?report=genbank&log$=nucltop&blast_rank=1&RID=0NCUGZ5801N) | 61523702 - 61523841 |
| TRINITY_GG_10824_c28_g1 | Lectin_C | 9.04233059 | 6.14E-37 | 1.10E-33 | [JAVIAB010000092.1](https://www.ncbi.nlm.nih.gov/nucleotide/JAVIAB010000092.1?report=genbank&log$=nucltop&blast_rank=1&RID=0NCUGZ5801N) | 76008067 - 76008327 |
| TRINITY_GG_28651_c10_g1 | Lectin_C | 8.95271527 | 1.14E-36 | 1.93E-33 | [JAVIAB010000124.1](https://www.ncbi.nlm.nih.gov/nucleotide/JAVIAB010000124.1?report=genbank&log$=nucltop&blast_rank=1&RID=0NCUGZ5801N) | 28793559 - 28793605 |
| TRINITY_GG_8506_c24_g1 | Lectin_C | 8.46824997 | 3.28E-35 | 4.16E-32 | [JAVIAB010000059.1](https://www.ncbi.nlm.nih.gov/nucleotide/JAVIAB010000059.1?report=genbank&log$=nucltop&blast_rank=1&RID=0NCUGZ5801N) | 62895895 - 62896011 |
| TRINITY_DN2216_c0_g2 | VWA | 11.4372746 | 3.80E-44 | 4.43E-40 | [JAVIAB010000088.1](https://www.ncbi.nlm.nih.gov/nucleotide/JAVIAB010000088.1?report=genbank&log$=nucltop&blast_rank=1&RID=0NDFMSR2013) | 14223378 - 14223634 |
| TRINITY_DN42977_c0_g1 | VWA | 10.4121983 | 4.62E-41 | 2.05E-37 | [JAVIAB010000088.1](https://www.ncbi.nlm.nih.gov/nucleotide/JAVIAB010000088.1?report=genbank&log$=nucltop&blast_rank=1&RID=0NDFMSR2013) | 13812368 - 13812634 |
| TRINITY_GG_8072_c1_g1 | VWA | 9.78808008 | 3.50E-39 | 1.12E-35 | [JAVIAB010000088.1](https://www.ncbi.nlm.nih.gov/nucleotide/JAVIAB010000088.1?report=genbank&log$=nucltop&blast_rank=1&RID=0NDFMSR2013) | 13788249 - 13788610 |
| TRINITY_DN19045_c0_g2 | VWA | 9.243237 | 1.53E-37 | 3.21E-34 | [JAVIAB010000008.1](https://www.ncbi.nlm.nih.gov/nucleotide/JAVIAB010000008.1?report=genbank&log$=nucltop&blast_rank=1&RID=0NDFMSR2013) | 2065959 - 2066431 |
| TRINITY_GG_8854_c1_g1 | VWA | 7.93364649 | 1.33E-33 | 1.14E-30 | [JAVIAB010000145.1](https://www.ncbi.nlm.nih.gov/nucleotide/JAVIAB010000145.1?report=genbank&log$=nucltop&blast_rank=1&RID=0NDFMSR2013) | 34700024 - 34702174 |
| TRINITY_DN5046_c0_g1 | VWA | 7.73669919 | 1.83E-27 | 4.50E-25 | [JAVIAB010000088.1](https://www.ncbi.nlm.nih.gov/nucleotide/JAVIAB010000088.1?report=genbank&log$=nucltop&blast_rank=1&RID=0NDFMSR2013) | 14139408 - 14139648 |
| TRINITY_GG_50378_c0_g1 | VWA | 7.60887454 | 2.57E-12 | 3.58E-11 | [JAVIAB010000092.1](https://www.ncbi.nlm.nih.gov/nucleotide/JAVIAB010000092.1?report=genbank&log$=nucltop&blast_rank=1&RID=0NDFMSR2013) | 66950606 - 66951536 |

Supplementary Table 11. 44 putative active peptides of antimicrobial and anticancer prediction

| SEQID | Sequence | Family | Antimicrobial | Anticancer |
| --- | --- | --- | --- | --- |
| TRINITY_DN7810_c1_g1_i3.p1 | QDELTLQLINCESVEITRLKPWQVKMKKERQIKHITDYKLINCESVKITRLKEWQVKMKKERQIKHITDYKLINCESVKITRLKEWQVKMKKERQIKHITDYTFEM | Transferrin | antimicrobial |  |
| TRINITY_DN69842_c0_g1_i1.p1 | ARPTRWCVFNNAEEIKCKQLKFELDKAKVTNATLKTLLPETFQCIRTFDIFACMALIERDEADLINLDSGLGYFSGRVHNMMPIMAEDYGYELKIRPSYYSVAMFKSTDQVTRENLKQKTVCFSTIGMSAGWVFPIGQLLQMNYIDVDQCNAIVKSVSSFFRGLCLPGSLTSFYNPFGNNPTSVCDLCTGKNEEFCATSDDYAGYDGAFKCMASGNGQLTFLRHDTIAQMTGTDNNQTGSGYLPENFRLLCPDGTTNTVDNYAACNWGEVTSNVIMTSAVRDPETVAGYKSFLRLLEQMFGPNGFSTSKFKLFRSETSYPETDVFKQTLTRKNLMFSDQTKKLMDIGTETYYTWVGEIFPTLLNEMNRCPSETIRWCLTSLAEKLKCEDMIMAFKAKDLRPEMDCLYGGNTTNCMDMIYRGDADLINLDAGDVYIAGRRYGLVPIIAEDYGDMTMQFKVVAAARKTDKYTTLFNMRGKRSCQPGINRGDGWVIPLNIFIETEQFLPTDCSIFRNIGELFARSCIPGALDTEYNPKVKPINLCEGCGGGGFRKCQRNGEEQYYGASGAFRCLVEKGGDVAFVRHLTVRDNTDGRNHAIWARNRRSDDYELMCKDGRRLNVDHFEECHLGYVPANVIVTAADMNENKKDIIWNLLNYGQQFFSSDIDGDFHMFDSGIWYTDLIFTDAAVRLIRIPEERRNYKDWLGESFLAQIENLHKYTCVNPDSGISLSPSLLLSLVVTLITSLLIQL | Transferrin |  | anticancer |
| TRINITY_DN18316_c0_g3_i8.p1 | FRITDIISCSSDDFRVTGIITCSPDDFRVTGIISCSSDGFRVTGIITCSSDDFRVTGIISCSSDEFRVTGIITCSSDEFRVTGIITCSSDDFRVTGIISCSSDTHIRFTLENRLITHFGTLQPRRLNSFHSFLSSLVSSPCLSRVLSIFPHALS | Tachystatin | antimicrobial |  |
| TRINITY_DN18316_c0_g3_i1.p2 | FRITDIISCSSDDFRVTGIITCSPDDFRVTGIISCSSDGFRVTGIITCSSDDFRVTGIISCSSDEFRVTGIITCSSDEFRVTGIITCSSDDFRVTGIISCSSDGFRVTGIITCSPDDFRVTGIISCSSDGFRVTGIITCSSDDFRVTGIISCSSDEFRVTGIITCS | Tachystatin | antimicrobial |  |
| TRINITY_GG_17506_c6_g1_i2.p1 | SDCVVLLCTAVTSDCVVLLCTTTTSDFRFFIHSKCATNNGSFLTRHSSFTPNVRPNNGSFLTRHSSFAPNVRPNDDYLPVRIGECGNKVRYN | Metchnikowin | antimicrobial | anticancer |
| TRINITY_DN36562_c0_g2_i2.p1 | FPLSCGLSSTLLSPSCERWSLQILAKCYLNVLAKCYLNVLSKCYLNVLSKCYLNVLAKCYLNVLSKCYLKVLSKCYIQHLILGLC | Maximin | antimicrobial |  |
| TRINITY_DN3477_c0_g1_i12.p3 | TQCVCSCYVNSMCVLLLCQRNVCAPVMSTQYVCSCYVNSMCVLLLCQRNVCAPVMSTQYVCASVMSNQCVCSCYVKSMCVLLLCQLAQCV | LEAP-2 | antimicrobial |  |
| TRINITY_DN3477_c0_g1_i1.p2 | TQCVCSCYVNSMCVLLLCQRNVCAPVMSTQYVCSCYVNSMCVLLLCQRNVCAPVMSTQYVCSCYVYSICVCFGYVKSMCVLLLCQINVCAPVMSTQCVCSCYGYCSTHANSQVVLWLWKARISCKRIKNP | LEAP-2 | antimicrobial | anticancer |
| TRINITY_GG_50904_c22_g1_i3.p1 | KVTAAKFHSSLDLYISVGEEWIPVPTSCKETFSCIVTLDRKSRELNPVVVNAEVNGLICDNTDEKDAVIYTDGSVRRHQRSQCMGIYSTLGRQNSQRRQWCIRYEHE | LEAP-2 | antimicrobial |  |
| TRINITY_GG_28651_c212_g1_i1.p2 | IIGCIFNSCEQVCDPVWQGIFLVPKCSCRSGYTISTHDKTKCTLDKPAITCPSGFIQVGSSCEDVNECADNTARCEQICS | LEAP-2 | antimicrobial | anticancer |
| TRINITY_DN15048_c0_g1_i2.p1 | DLASCINTIQSEVQNGDINCDNIETYTKCVLRETGFSNPVMATKSNLHKMSEKIRLVMVRFGLSCDINVRNIVKQLQEEESHNGNDASTPSRYEDATTINECLKVFTLHHSLNCSEFIPYIHCILRVSKLNKLDSDTRERFYKNYQAKINTILAHHGLKCDIDIKAIENQIRREQNEEDESVTMADKTAQEQNEGDRSVPMADKTAQEQNEGDRSVPMADKTAQEQNEGD | Halocidin | antimicrobial |  |
| TRINITY_DN15048_c0_g1_i8.p1 | DLASCINTIQSEVQNGDINCDNIETYTKCVLRETGFSNPVMATKSNLHKMSEKIRLVMVRFGLSCDINVRNIVKQLQEEESHNGNDASTPSRYEDATTINECLKVFTLHHSLNCSEFIPYIHCILRVSKLNKLDSDTRERFYKNYQAKINTILAHHGLKCDIDIKAIENQIRREQNEEDESVTMADKTAQEQNEGDRSVPMADKTAQEQNEGDRSVPMADKTAQEQNEGDRSVPMADKRAQEQTKEDGSGASYRPFPLITLIMSIVMSVVVLY | Halocidin | antimicrobial |  |
| TRINITY_GG_45850_c0_g4_i1.p1 | DLASCINTIQSEVQNGDINCDNIETYTKCVLRETGFSNPVMATKSNLHKMSEKIRLVMVRFGLSCDINVRNIVKQLQEEESHNGNDASTPSRYEDATTINECLKVFTLHHSLNCSEFIPYIHCILRVSKLNKLDSDTRERFYKNYQAKINTILAHHGLKCDIDIKAIENQIRREQNEEDESVTMADKTAQEQNEGDRSVPMADKRAQEQTKEDGSGASYRPFPLITLIMSIAMSVIVLY | Halocidin | antimicrobial |  |
| TRINITY_GG_45850_c0_g4_i3.p1 | DLASCINTIQSEVQNGDINCDNIETYTKCVLRETGFSNPVMATKSNLHKMSEKIRLVMVRFGLSCDINVRNIVKQLQEEESHNGNDASTPSRYEDATTINECLKVFTLHHSLNCSEFIPYIHCILRVSKLNKLDSDTRERFYKNYQAKINTILAHHGLKCDIDIKAIENQIRREQNEEDESVTMADKTAQEQNEGDRSVPMADKTAQEQNEGDRSVPMADKRAQEQTKEDGSGASYRPFPLITLIMSIVMSVVVLY | Halocidin | antimicrobial |  |
| TRINITY_GG_43926_c2_g1_i1.p1 | DLASCINTIQSEVQNGDINCDNIETYTKCVLRETGFSNPVMATKSNLHKMSEKIRLVMVRFGLSCDINVRNIVKQLQEEESHNGNDASTPSRYEDATTINECLKVFTLHHSLNCSEFIPYIHCILRVSKLNKLDSDTRERFYKNYQAKINTILAHHGLKCDIDIKAIENQIRREQNEEDESVTMADKTAQEQNEGGRSVTMADKTAQEQNEGDRSVPMADKTAQEQNEGGRSVTMADKTAQEQNEGDRSVPMADKRAQEQTKEDGSGASYRPFPLITLIMSIVMSVVVLY | Halocidin | antimicrobial |  |
| TRINITY_DN47286_c0_g1_i1.p1 | QILENLHSNSDAFSSVSVVIDAVAVNIFCTASCKNWLLIPCSTLEPVFVAFNLLTFLQLFPLIIIIIIKHQACWHR | Gaegurin |  | anticancer |
| TRINITY_DN33944_c0_g1_i2.p1 | QKHHRHIIYYHKHTTDTSSTITKTSQIHYLLSQAHHRYIIYYHKNITDTSSTITSTPQIHHLLSQKHHRYIIYYHKNITDTLSTITSTPPIHHLLSQKHHRYIIYYHKHNTETSSTITK | Defensin | antimicrobial | anticancer |
| TRINITY_DN34357_c0_g1_i6.p4 | YCPTQCYCLHSKLLPPLNVIASTQCYCIHSKLLPPLKVIASTQSYCLHSMLLPPLKVIAPLNVTASTQSYCLHSKLLPPINVTASTQYY | Defensin | antimicrobial |  |
| TRINITY_DN56294_c0_g1_i7.p3 | VNLCSNCQSMFKPSIYVQTVNLCSNCQSMFKPSIYVQTVNLCSNCPSMFKLSIYVQTVNLCSNRQSMFKLSIYVQTVNLCSNCQSMFKLSIDVHIND | Defensin |  | anticancer |
| TRINITY_DN70238_c0_g1_i1.p1 | YQVKRKTFIRIEEVSALESSVKETLEYVTDEYNKKSEDLYNFRILRILKIMKQVTGHLEYHITVEMQRTTCLKTETSLCDIQKGELHKKIQCYFSVYAILWVEVFKILKKN | Cystatin | antimicrobial |  |
| TRINITY_DN43979_c0_g1_i1.p3 | SSPGKPPRLVGGPMDASVEEEGVRRALDFAVGEYNKASNDMYHSRALQVVRARKQIVAGVNYFLDVELGRTTCTKTQPNLDNCPFHDQPHLKRKAFCSFQIYAVPWQGTMTLSKSTCQDARSEERRVGKECNCRCRSRWS | Cystatin | antimicrobial |  |
| TRINITY_DN154973_c0_g1_i1.p1 | VGVDQSKNEVKAQNYFGSINISNANVKQCVWFAMKEYNKESEDKYVFLVDKILHAKLQITDRMEYQIDVQISRSNCKKPLNNTENCIPQKKPELEKKMSCSFLVGALPWNGEFNLLSKECKDV | Cystatin | antimicrobial |  |
| TRINITY_GG_45872_c0_g1_i1.p1 | LEKNLLFNNNNARHRREGLSSNFPDLPEYSPQRTGGYQEVNLSALYGKDRASFKNAAKQAIRLMNAVKTENGSNNNMPLKLKKINAVATQLVAGTNYRYVLTLIIDGKKRICRVKVSHLQPWIKGGTWSICVVASNDPCKLFASPCRKPRSVSLLGGESAVDANDAMVQKAAQFAVEHVNKMSNSLFMQGLFSVTHATKQIINGIRYHLHLKTVFTSCRNKPENHHKTLDECPAAADSKPQECDISVIYSQGAYTMEKFQCTPLKNIIGGDDHDYVPHVKRALNVKQMSVLDGDAIKYTPTSDEKCSQYLNAFTEFKTKHSRVYESAEKENFRFQIFCNNMEKVKIIQAVEQGSAKYGATQFADLSEEEFKKQYLGFKLPLTQKRQWPQAKIPDGPIPESWDWRNHNAVTPVKNQGWCGSCWAFSTTGNIEGQWAIHSKSQNLLSLSEQELVDCDKIDQGCNGGYMYDAYEALMEIGGIETENDYKYTGQDQKCQFNKSEVVVKVTGAVNISKDEGEMAAWLYKNGPIAIGINAFPMQFYLWGIAHPWKFFCDPNALDHGVLIVGYGKQGREPYWIIKNSWGPKWGREGYYYIYRGDGSCGLNTMCSSATVA | Cystatin |  | anticancer |
| TRINITY_GG_45872_c0_g1_i2.p1 | LEKNLLFNNNNARHRREGLSSNFPDLPEYSPQRTGGYQEVNLSALYGKDRASFKNAAKQAIRLMNAVKTENGSNNNMPLKLKKINAVATQLVAGTNYRYVLTLIIDGKKRICRVKVSHLQPWIKGGTWSICVVASNDPCKLFASPCRKPRSVSLLGGESAVDANDAMVQKAAQFAVEHVNKMSNSLFMQGLFSVTHATKQIINGIRYHLHLKTVFTSCRNKPENHHKTLDECPAAADSKLKNIIGGDDHDYVPHVKRALNVKQMSVLDGDAIKYTPTSDEKCSQYLNAFTEFKTKHSRVYESAEKENFRFQIFCNNMEKVKIIQAVEQGSAKYGATQFADLSEEEFKKQYLGFKLPLTQKRQWPQAKIPDGPIPESWDWRNHNAVTPVKNQGWCGSCWAFSTTGNIEGQWAIHSKSQNLLSLSEQELVDCDKIDQGCNGGYMYDAYEALMEIGGIETENDYKYTGQDQKCQFNKSEVVVKVTGAVNISKDEGEMAAWLYKNGPIAIGINAFPMQFYLWGIAHPWKFFCDPNALDHGVLIVGYGKQGREPYWIIKNSWGPKWGREGYYYIYRGDGSCGLNTMCSSATVA | Cystatin |  | anticancer |
| TRINITY_DN14900_c0_g1_i5.p2 | YMFGCQCYSPVVGYMFGCQCYSPVVGYMFGSQCYSPEVGYMFGCQCYSPVVGYIFWTSVLQSCSKLYVWMSVLQSCSRLHVWMSVLQSCSRLHVWISVLQSCSRLHVWMSVLQSCSRLYVWISVLQSCSRLYILD | Cyclotide |  | anticancer |
| TRINITY_DN31243_c0_g1_i2.p2 | QQNLLHTYHIYKASLLYVFSCVSIDCHSHQNLHHTHHIYKASLLYVFSCVSIDCHSHQNLHHTHHIYKASLLYVFSCVSIDCYYQQILHHTHHTCKASLQIGRAHV | Cyclotide | antimicrobial | anticancer |
| TRINITY_DN27619_c0_g1_i16.p3 | AHVVRCLITFSSCGQMSHYIQLMWSDVSLHSAHVVRCLITFNSCGQMSHYIQLMWSDVSLHSAHVVRCLITFSSCGQMSHYIQLMWSDVSLHSAHVVGCLITFNSCGQMSHYIQLMWSDVS | Brevinin |  | anticancer |
| TRINITY_GG_9208_c33_g1_i1.p3 | EQIPYHKYRTCKVSLLYVFFYVFANCCCEQIPYHKYRTCKVSLLYVLFCVYSGHCSDQILLHRYHTCKVSCQCVSFYISQDC | Brevinin | antimicrobial | anticancer |
| TRINITY_GG_60690_c0_g1_i2.p2 | LEVLSTVGSAICCQISGSAIYCQISESAIFCWKCYLLSNLWKCYLLLEVLSTVKSLEVLSTVGSAICCQISGSAIFCWKCYLLSNL | Bombinin | antimicrobial |  |
| TRINITY_DN65312_c0_g4_i1.p2 | QKNMHHKHHICRASLLYVFFCVSIDCHFQKSMYHKHHICRASLQYVFFCVSIDCHSEQSMYHKDHICKASLLCASSCVLLDCL | Bacteriocin | antimicrobial | anticancer |
| TRINITY_DN47152_c0_g1_i4.p3 | GSSFDEGRIDGGSSIVGGGSIDAGSSIDASCSIDAGSSIDDGGSSIDGGCSIDAGCSIDGGCSIDAGSIDAGSSIDEGCSIDEVVVLMQVVLMQVVVLMKVVVLMEVVVLMQVKVVVLMRL | Bacteriocin | antimicrobial |  |
| TRINITY_DN29123_c1_g1_i1.p2 | QSQTCYSPSVGRSRTPVAHQVLVTVANLLLTKCWSQSHTCCSPSVGHSRKPVTHQVLVAVAHLLLTKCWSQSHTCYSPSVGHSRKPVTHQVLVAVAHLLLTKCWSQSHTCCSLSVASSRPAPCRG | Bacteriocin | antimicrobial |  |
| TRINITY_DN1185_c0_g2_i20.p2 | QDNTTAYPCDEICLNGGSCTVENDKPTCDCQVGFSGENCESSAHPGICPNIEENSMGVCGGNVIARKDSLEKDVRAQLILGHVPTLRKIPWVCVVVSCVHQMRTVPLTRSVANHQNVIARKDSLEKDVRAQLILGHVPTLRKIPWVCVVVSCVHQMRTVP | Bacteriocin | antimicrobial | anticancer |
| TRINITY_DN3371_c0_g2_i5.p1 | NIISVCTHHILEKTVCTPNLSEKMVCTPNLLEKTVCTPNLLEKTVCTPNLLEKTVCTPHLPKKTVCTHYLSEKKMCTHYLSEKTMCTHHLSEKTMYTHHLSEKNNYLSLIQTYIRFCRQ | Bacteriocin | antimicrobial |  |
| TRINITY_DN3371_c0_g2_i8.p1 | NIISVCTHHILEKTVCTPNLSEKMVCTPNLLEKTVCTPNLSEKMVCTPNLLEKTVCTPNLLEKTVCTPNLLEKTVCTPHLPKKTVCTHYLSEKTMCTHHLSEKTMYTHHLSEKNNYLSLIQTYIRFCRQ | Bacteriocin | antimicrobial |  |
| TRINITY_DN3371_c0_g2_i7.p1 | NIISVCTHHILEKTVCTPNLSEKMVCTPNLLEKTVCTPNLLEKTVCTPNLLEKTVCTPHLPKKTVCTHYLSEKTMCTHHLSEKTMYTHHLSEKNNYLSLIQTYIRFCRQ | Bacteriocin | antimicrobial |  |
| TRINITY_DN1809_c0_g4_i6.p1 | NIISVCTHHILEKTVCTPNLSEKMVCTPNLLEKTVCTPNLLEKTVCTPNLSEKMVCTPNLLEKTVCTPNLLEKTVCTPNLLEKTVCT | Bacteriocin | antimicrobial |  |
| TRINITY_DN16689_c0_g1_i3.p1 | CSSLQHKSSACYSLQHNSSACYSLQYNSSACSSLQHNFSACFSLQHNFSACSSLQHNFSACFSLHYNSSECSSLQHNFSACSSLQHKSSACYSLQHNSSACYLLQYNSSACSSLQHNFSACSSLQHNFSACFSLHYNSSECSSLQHNFSACSSLQHKSSACYSLQHNSSACYLLQYNSSAC | Bacteriocin | antimicrobial |  |
| TRINITY_GG_37343_c0_g1_i6.p2 | HLILSPATVTWHCHMPPSPLPLSPATGTCHCHLSLSPVICLCHLPLSPATVTCHRHLLLSPATGTCHCHLSLSPVICLCHLPLSPATVTCHRHLLLSPTTGTCHCHLSLSPVICHCHLPLSHATVTCYYHLPLAPAIVTYHSHLLSATVTCHCHLPL | Bacteriocin | antimicrobial |  |
| TRINITY_GG_931_c1_g1_i4.p1 | DVNLEYTSVINHLKRVSKNDVIPIPENESGPVNVTISVSLVNIIDVDVKRDEVEVLIWLTLAWENKALAWDLKEEKLNHSDVRVPANYLWTPDIVTVNNIKKQEDLSPNLALVDNTGLVHWVRFLPIRVSCDFSNLNHQISSVNCTLKFSSWVYSGNLLTISPDSAFDRNEYVENPKYDLGNIAHTKHITYYASSPEIPYEDFRTNFTIKSTYRSVLSQYE | Bacteriocin | antimicrobial |  |
| TRINITY_GG_931_c1_g1_i3.p1 | DVNLEYTSVINHLKRVSKNDVIPIPENESGPVNVTISVSLVNIINVDSDRNQIEVLIWLGLQWRNKALAWDMKEEKLNHGDVTVPANYLWTPDIIPVNNIKKQEDLSPNLALVDNTGLVHWVRFLPIRVSCDFSNLNHQISSVNCTLKFSSWVYSGNLLTISPDSAFDRNEYVENPKYDLGNIAHTKHITYYASSPEIPYEDFRTNFTIKSTYRSVLSQYE | Bacteriocin | antimicrobial |  |
| TRINITY_DN4368_c0_g2_i5.p1 | HIVHYCRSYCTCHMEHYCSHIDCVIIAGHMEHYCSHIVLLLQVTWNTIPGHIVLLLQVTWNTIAVTLCCYCRSHGTLFQVTLCYYCRSHGTLYTIAGHIAHYCSHIVSLLQVTLYTIAGHIAHYCSHIVSLLQ | Ascaphin | antimicrobial |  |
| TRINITY_GG_31860_c1_g1_i1.p2 | IGLQQTLCKPSCFLPFFLDHFISPPHPLHLCFHLSSPSIFWPYPLYPSHPLHLCFHLSSPSIFWPYPLYPPLEFQNRT | Abaecin | antimicrobial |  |
| TRINITY_GG_56694_c0_g1_i5.p3 | LSCHCHGPFSFVDSSFQLSSTICPVTVVVPFPLWTHPSNYLQQSVLSLSWSLFLCGLIFPTIFNSLSCHCHGPFSFVDSSFQLSSTVCPVTVVVPFPLWTHPSNYLQQSVLSLSWSFQLSSTIC | Abaecin | antimicrobial |  |
