# Supplementary material for: De novo genome assembly and transcriptome sequencing in foot and mantle tissues of Megaustenia siamensis reveals components of adhesive substances
Source: Sci Rep. 2024 Jun 14;14:13756. doi: 10.1038/s41598-024-64425-6 (PMC11178922; doi:10.1038/s41598-024-64425-6)
Supplement: Supplementary file 2 — Supplementary Figures. [file 41598_2024_64425_MOESM2_ESM.docx]

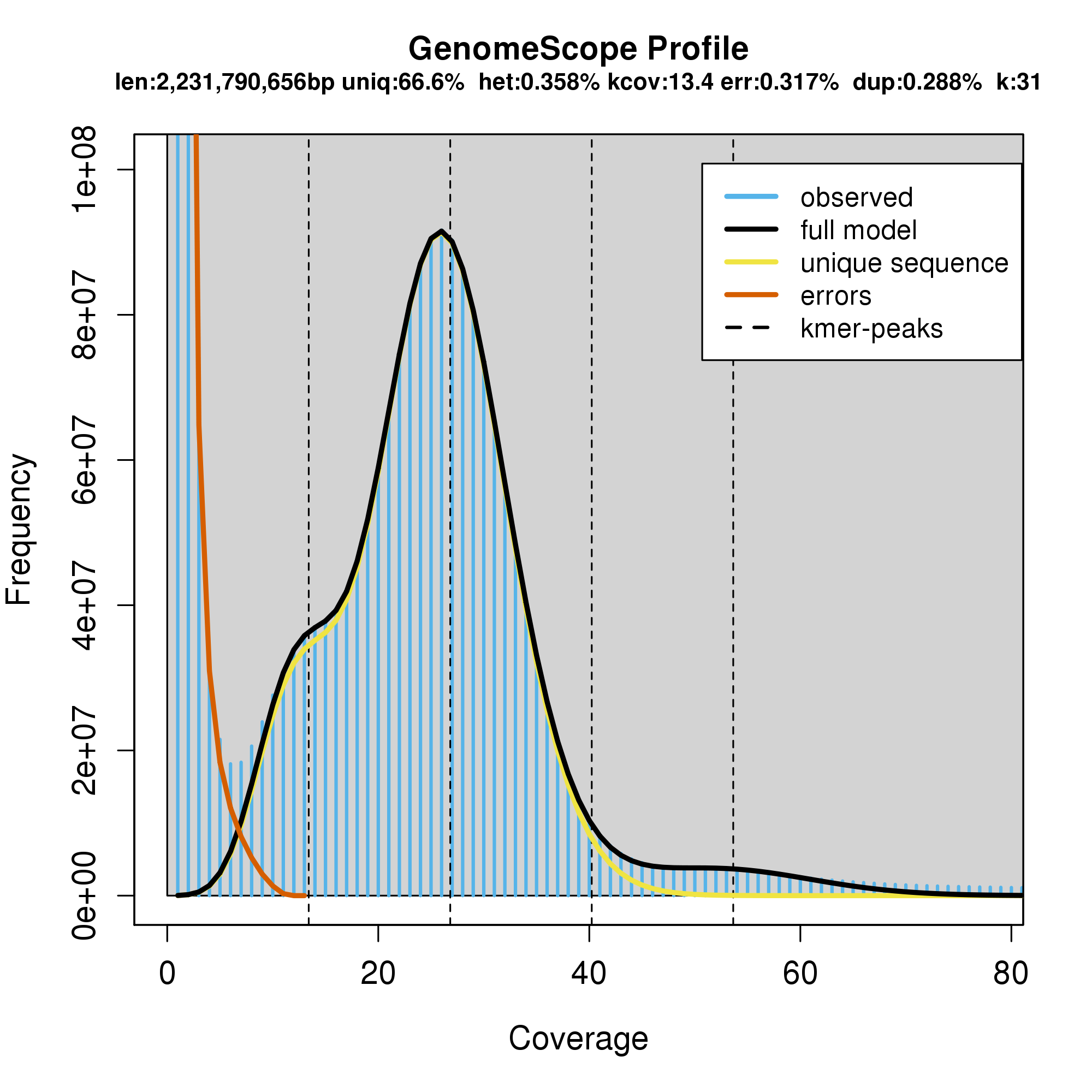


Supplementary Figure 1. GenomeScope estimation of genome size of *M.siamensis* based on the distribution of 31-mer in the illumina sequencing data


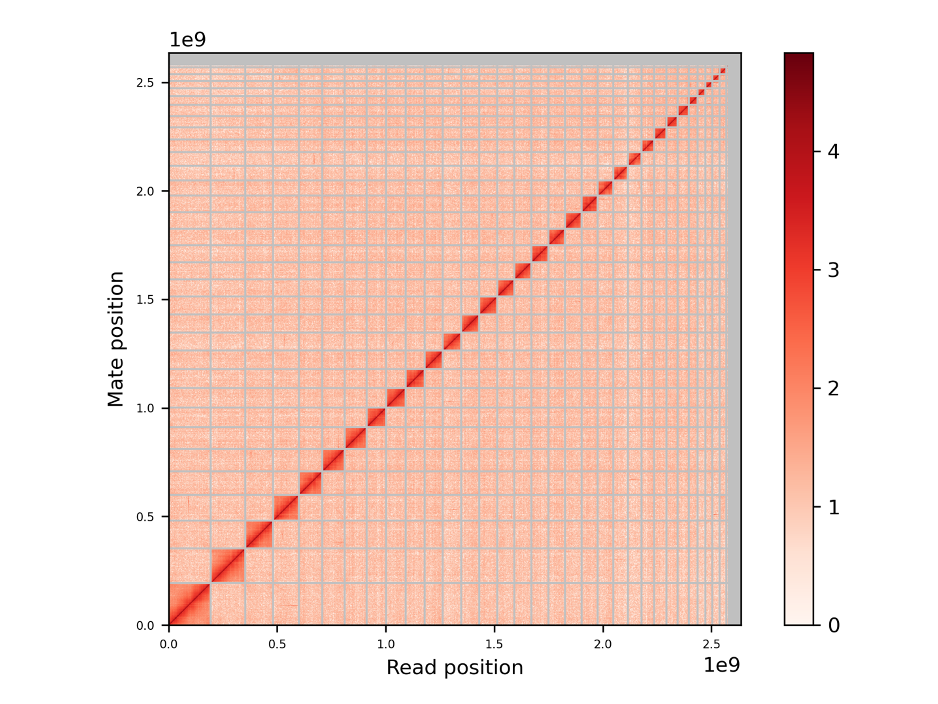


Supplementary Figure 2. Hi-C linkage density histogram of *M.siamensis*. The x and y axes in this histogram show the mapping positions of the first and second read in a read pair, respectively, grouped into bins. The color bar indicates contact density from red (high) to white (low)
